# Novel mannosylerythritol lipid biosurfactant structures from castor oil revealed by advanced structure analysis

**DOI:** 10.1093/jimb/kuab042

**Published:** 2021-07-29

**Authors:** Alexander Beck, Fabian Haitz, Isabel Thier, Karsten Siems, Sven Jakupovic, Steffen Rupp, Susanne Zibek

**Affiliations:** Institute of Interfacial Process Engineering and Plasma Technology IGVP, University of Stuttgart, 70569 Stuttgart, Germany; Fraunhofer Institute for Interfacial Engineering and Biotechnology IGB, 70569 Stuttgart, Germany; Fraunhofer Institute for Interfacial Engineering and Biotechnology IGB, 70569 Stuttgart, Germany; AnalytiCon Discovery GmbH, 14473 Potsdam, Germany; AnalytiCon Discovery GmbH, 14473 Potsdam, Germany; AnalytiCon Discovery GmbH, 14473 Potsdam, Germany; Institute of Interfacial Process Engineering and Plasma Technology IGVP, University of Stuttgart, 70569 Stuttgart, Germany; Fraunhofer Institute for Interfacial Engineering and Biotechnology IGB, 70569 Stuttgart, Germany; Institute of Interfacial Process Engineering and Plasma Technology IGVP, University of Stuttgart, 70569 Stuttgart, Germany; Fraunhofer Institute for Interfacial Engineering and Biotechnology IGB, 70569 Stuttgart, Germany

**Keywords:** MEL, Ustilaginaceae, Fungi, Ricinoleic acid, Hydroxy fatty acid, LC-MS, MALDI-TOF-MS

## Abstract

Mannosylerythritol lipids (MELs) are glycolipid biosurfactants produced by fungi of the *Ustilaginaceae* family in the presence of hydrophobic carbon sources like plant oils. In the present study, we investigated the structural composition of MELs produced from castor oil using seven different microorganisms and compared them to MEL structures resulting from other plant oils. Castor oil is an industrially relevant plant oil that presents as an alternative to currently employed edible plant oils like rapeseed or soybean oil. The main fatty acid in castor oil is the mono-hydroxylated ricinoleic acid, providing the possibility to produce novel MEL structures with interesting features. Analysis of the produced MELs from castor oil by different chromatographic and mass spectrometry techniques revealed that all seven microorganisms were generally able to integrate hydroxylated fatty acids into the MEL molecule, although at varying degrees. These novel MELs containing a hydroxy fatty acid (4-*O*-[2′-*O*-alka(e)noyl-3′-O-hydroxyalka(e)noyl-4′/6′-*O*-acetyl-β-*D*-mannopyranosyl]-erythritol) were more hydrophilic than conventional MEL and therefore showed a different elution behavior in chromatography. Large shares of novel hydroxy MELs (around 50% of total MELs) were found for the two MEL-B/C producing species *Ustilago siamensis* and *Ustilago shanxiensis*, but also for the MEL-A/B/C producer *Moesziomyces aphidis* (around 25%). In addition, tri-acylated hydroxylated MELs with a third long-chain fatty acid esterified to the free hydroxyl group of the hydroxy fatty acid were identified for some species. Overall, production of MEL from castor oil with the investigated organisms provided a complex mixture of various novel MEL structures that can be exploited for further research.

## Introduction

Mannosylerythritol lipids (MELs) are glycolipid biosurfactants produced by fungi of the *Ustilaginaceae* family. They have been receiving increasing attention due to their interesting phase-behavior and self-assembling properties (Worakitkanchanakul et al., 2009), cell-differentiation activities in mammalian cells (Isoda et al., 1997; Wakamatsu et al., 2001), and protein or antibody interaction (Konishi et al., 2007). Moreover, possible applications of MELs have been reported in the field of hair and skin care due to their moisturizing effect and ceramide-like function (Morita et al., 2009, 2010; Yamamoto et al., 2012), or as surface modifiers in bioplastics (Fukuoka et al., 2018), just to name a few examples.

MELs consist of a hydrophilic sugar core, 4-O-β-D-mannopyranosyl-D-erythritol, and multiple hydrophobic residues which usually include two fatty acid chains at C2′ and C3′ and a variable degree of acetylation at C4′ and C6′ of the mannose part. Classically, MELs are distinguished by their acetylation pattern into the congeners MEL-A, -B, -C and –D; where MEL-A is di-acetylated, MEL-B and -C are mono-acetylated at C6′ and C4′, respectively, and MEL-D is not acetylated (Kitamoto et al., 1990). Their polarity is hence increasing from MEL-A to MEL-D. Additionally, MEL can be classified according to the number and length of the fatty acid chains. Common MELs are di-acylated, containing two fatty acid residues at C2′ and C3′. Besides these di-acylated MELs, there have also been reports of mono- and tri-acylated MELs depending on process conditions (Fukuoka et al., 2007a, 2007b). The chain length and combination of the two fatty acid residues in di-acylated MELs are species specific and can range from C_2_ and C_4_ up to C_16_ or even C_18_ fatty acids. In combination, the two fatty acids usually add up to a total of 18–22 carbon atoms, yielding di-acylated MELs with a molecular weight between 550–700 Da (Beck et al., 2019).

In a previous work, we had characterized the influence of different producer species and common oil substrates like soybean, rapeseed, or olive oil on the chemical structure of the produced MEL mixtures in detail (Beck et al., 2019). It had been shown that different species produced a different MEL product and that substrate oils could influence the degree of saturation in fatty acid side chains. With the current work, we want to fill the remaining gaps using a substrate oil with an unconventional fatty acid composition, namely castor oil. Castor oil is a vegetable oil that is produced by pressing the seeds of the *Ricinus communis* plant. Annual production of castor oil accounts to around 0.6 million metric tons (Chauke et al., 2019), where the major share is originating from India. Interestingly, the triglycerides in castor oil contain up to 90% ricinoleic acid (12-hydroxy-9-cis-octadecenoic acid)—a mono-unsaturated fatty acid with a free hydroxyl group—along with minor amounts of oleic and linoleic acid (Mutlu & Meier, 2010). The specific chemical composition of ricinoleic acid with its functional hydroxyl group, which is not found in other commercially relevant vegetable oils, increases the polarity and presents a good starting point for many chemical derivatization reactions (Ogunniyi, 2006). As such, castor oil is a very interesting substrate for many industrial applications; for example, in the production of biodiesel, polymer materials, lubricants, paints, dyes, coatings, pharmaceuticals, cosmetics, or perfumes (Mutlu & Meier, 2010; Ogunniyi, 2006; Patel et al., 2016).

Castor oil, or ricinoleic acid respectively, has previously been reported for the production of microbial glycolipids as well, namely for sophorolipids (Bajaj & Annapure, 2015; Bhangale et al., 2014), MELs (Yamamoto et al., 2013) and enzymatically synthesized glycolipids (Konishi et al., 2011). In the work of Yamamoto et al. (2013), the authors were able to produce a novel MEL-B glycolipid containing a hydroxy fatty acid with the strain *Pseudozyma tsukubaensis* NBRC1940. The novel MEL-B, which contained mostly a saturated C_8:0_ and a C_14:1-OH_ hydroxy fatty acid, showed a more hydrophilic nature and a five-time higher critical micelle concentration (cmc) than corresponding MEL-B without hydroxy fatty acids. Other tested MEL producers like *Moesziomyces antarcticus* T-34, *Sporisorium graminicola* CBS10092, and *Pseudozyma hubeiensis* KM-59 did not show novel MEL structures from castor oil. Instead, they produced only structures similar to conventional MELs (Yamamoto et al., 2013).

In order to understand how castor oil can influence the resulting MEL structures, it is important to take into account the metabolic pathway of MEL production. The gene cluster responsible for MEL production comprises five essential genes—namely, *emt1, mac1, mac2, mat1*, and *mmf1*—which have first been discovered in *Ustilago maydis* (Hewald et al., 2006). Since then, the genes have been shown to be present in other MEL producing organisms like *M. antarcticus* T-34 and JCM10317 (Morita et al., 2013; Saika et al., 2014), *Moesziomyces aphidis* DSM70725 (Lorenz et al., 2014), *P. hubeiensis* SY62 (Konishi et al., 2013), *P. tsukubaensis* NBRC1940 (Saika et al., 2016), and lately, *S. graminicola* CBS10092 (Solano-Gonzalez et al., 2019) and *Ustilago hordei* Uh4857-4 (Deinzer et al., 2019). The respective enzymes include an erythritol/mannose transferase (Emt1), two acyltransferases (Mac1 and Mac2), an acetyltransferase (Mat1), and a putative transporter protein (Mmf1). For efficient MEL production, it is advantageous to supply both hydrophilic and hydrophobic precursor molecules to the culture. The mannosylerythritol sugar core is generated by glycosidic linkage of the two sugar precursors, mannose and erythritol, catalyzed by Emt1 (Hewald et al., 2006). The two fatty acids necessary for the hydrophobic part of MEL have been shown to be derived from a so-called chain-shortening pathway (Kitamoto et al., 1998). This pathway, which is localized in cellular peroxisomes (Freitag et al., 2014), employs a partial β-oxidation to produce medium-chain fatty acids with the preferred chain length for integration into MEL. The chain length is strain specific and is most probably governed by substrate specificity of the two acyltransferases Mac1 and Mac2 (Becker et al., 2021; Deinzer et al., 2019). Hence, when plant oils are used as substrate, the triglycerides are first hydrolyzed into free fatty acids and glycerol by action of a microbial lipase. The fatty acids are then imported into the cells, activated and directed to the chain-shortening pathway within peroxisomes. There they get attached to the mannosylerythritol core by the two acyltransferases Mac1 and Mac2, which have been shown to be co-localized in peroxisomes as well (Freitag et al., 2014). After selective acetylation by Mat1 to the respective MEL-A, -B, -C, or -D congeners, the MELs are ultimately exported out of the cell by the transporter protein Mmf1.

**Fig. 1.**
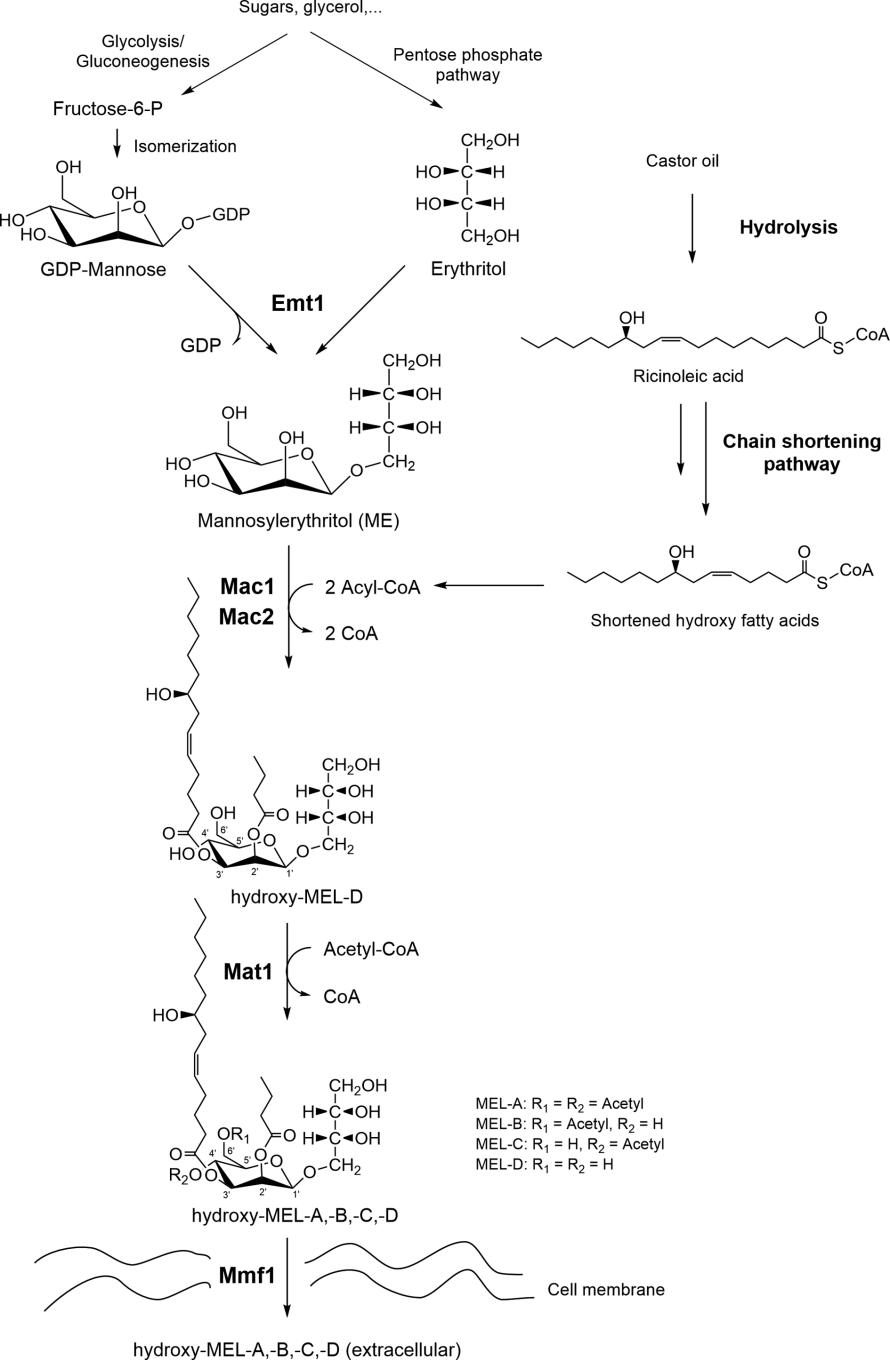
Proposed metabolic pathway for MEL production from castor oil. Chain-shortened hydroxy fatty acids from castor oil get esterified at C3′ of the mannose moiety by action of an acyltransferase.

Owing to the described fatty acid integration pathway, a structural correlation should exist between the fatty acids that are supplied to the culture in the form of plant oils and the structure of fatty acid side chains found in MEL. Our hypothesis was therefore that MELs produced from castor oil should possess hydroxy fatty acids as side chains (Fig. 1). The aim of this study was to assess if and to which degree seven selected *Ustilaginaceae* fungi were able to integrate hydroxylated fatty acids from castor oil into their MELs. Using several chromatographic and mass spectrometric techniques, we were able to resolve the resulting MEL structures in previously unknown detail and could show that *Ustilago siamensis, Ustilago shanxiensis*, and *M. aphidis* were promising producers of novel MELs with hydroxylated fatty acids from castor oil under the employed conditions.

## Materials and Methods

### Chemicals

All chemicals used in this work were obtained from either Merck (previously Sigma-Aldrich, Darmstadt, Germany), Carl Roth (Karlsruhe, Germany), VWR (Bruchsal, Germany), or Th. Geyer (Renningen, Germany) unless stated otherwise.

### Microorganisms and Substrates

For the reported experiments, seven fungal microorganisms of the *Ustilaginaceae* family, which had previously been studied for MEL production from other plant oils (Beck et al., 2019), were selected. *Moesziomyces parantarcticus* CBS 10005, *P. hubeiensis pro tem*. CBS 10077, *P. tsukubaensis pro tem*. CBS 422.96, *S. graminicola* CBS 10092, *U. siamensis* CBS 9960, and *U. shanxiensis* CBS 10075 were obtained from the Westerdijk Fungal Biodiversity Institute (CBS-KNAW; Utrecht, Netherlands). *M. aphidis* DSM 70725 was obtained from the German Collection of Microorganisms and Cell Cultures GmbH (DSMZ; Braunschweig, Germany). Cryoconserved cultures of the microorganisms were kept at −80°C in glycerol and thawed only once for immediate use. Stock cultures were plated on potato dextrose agar plates and incubated at 30°C for 3 days.

Castor oil (No. 259853, Sigma Aldrich/Merck, Germany) was used as the lipid substrate for MEL production.

### Cultivation Conditions

All cultivation conditions were the same as described previously in Beck et al. (2019). The culture medium for seed and main culture was a complex growth medium with the following composition: 30 g/l glucose, 3 g/l NaNO_3_, 0.2 g/l MgSO_4_*7H_2_O, 0.2 g/l KH_2_PO_4_, 1 g/l yeast extract, initial pH 6. Glucose was autoclaved separately from the rest of the medium and added aseptically. Plant oils for MEL production in the main culture were autoclaved and added aseptically, too.

Seed culture was performed in two steps in 15 ml glass tubes and 100 ml baffled shaking flasks with a working volume of 6 ml and 20 ml, respectively. Main cultures were carried out in 1 l shaking flasks with four baffles and a working volume of 200 ml. The first seed culture was inoculated with a full loop from the agar slant and incubated for 4 h at 30°C and 200 rpm to ensure homogenization of the culture. The second seed culture was inoculated with 1–2 ml of the first preculture to a defined optical density of 0.05 at 625 nm (OD_625_) and incubated for 24 h at 30°C and 110 rpm. The main culture was inoculated with 10–20 mL of the second seed culture to an initial OD_625_ of 0.3. The initial growth phase on glucose as sole carbon source took place for 72 h, until the cells reached their stationary phase due to substrate limitation. Then 8% (v/v) castor oil was fed to start MEL production. The full cultivation process was maintained for 504 h (21 days) to obtain high MEL yields. All cultivations were performed in independent triplicates at 30°C and 110 rpm in an orbital shaker (shaking diameter d = 50 mm).

Samples were taken intermittently during growth and production phase to follow growth, substrate consumption, and product formation. MEL formation from castor oil was occasionally assessed by HPTLC after extraction of culture broth samples with ethyl acetate (1:1 v/v). The organic phase obtained was separated, collected, and dried. The raw extract was then resuspended in ethanol to a concentration of 20 g/l and spotted onto HPTLC silica 60 plates (20×10 cm, Merck, Germany) using the ATS4 automatic TLC sampler (CAMAG, Muttenz, Switzerland). The plates were developed in chloroform-methanol (80:12, v/v) and stained with an acetic acid/*p*-anisaldehyde/sulfuric acid (97:1:2 v/v) reagent solution. Pure castor oil, hydrolyzed castor oil, and ricinoleic acid (90% purity) were used as reference materials to verify the peak pattern.

### Extraction and Chromatographic Purification of MEL for Structure Analysis

When the cultivations were terminated after 21 days, the entire culture broth of 200 ml was extracted two times with an equal volume of technical ethyl acetate by shaking at 110 rpm for 10 min. After separation of the aqueous and organic phases by centrifugation, the organic phases were combined, dried with anhydrous sodium sulfate, and filtered. Finally, the solvent was removed with a rotary evaporator at low pressure (p = 25 mbar, T = 50°C).

The raw MEL extract still contained unconsumed acylglycerides, free fatty acids, and other hydrolysis products from the castor oil substrate and was further purified by flash chromatography on silica gel (60 Å, 60–200 μm, Merck, Germany) in a packed glass column (44×5 cm, Büchi, Switzerland). For this, the raw extracts from the biological triplicates were combined, dissolved in 500 ml n-heptane-isopropanol (8:2, v/v), and applied to the column at a flow rate of 50 ml/min. Acylglycerides and free fatty acids were then eluted with 3 L n-heptane-isopropanol (8:2, v/v) at 70 ml/min, before the purified MEL fraction was eluted with 2 l technical ethanol at 45 ml/min. After complete evaporation of ethanol in the rotary evaporator at low pressure (p = 25 mbar, T = 50°C), the purified and highly viscous MEL was obtained. The low pressure of 25 mbar was necessary to ensure full removal of residual ethanol from the highly viscous MEL. These purified MELs were then used for structural analysis.

From the purified MEL of *U. siamensis* with castor oil, 0.75 g were additionally separated by preparative HPLC SEPBOX (Sepiatec, Germany) on a 250×50 mm column filled with LichrospherSelect B, 10 μm (Merck) at a flowrate of 80 ml/min with the following gradient: Solvent A: 5 mmol ammonium formate buffer adjusted with formic acid to pH 3, solvent B: 5 mmol ammonium formate buffer in methanol-acetonitrile 1:1 (v/v) gradient 52% A to 100% A in 120 min. Fractions were collected every 30 s and analyzed by liquid-chromatography with mass spectrometry (LC-MS). Fractions containing pure MEL were combined, evaporated, and analyzed by LC-MS and nuclear magnetic resonance (NMR).

### Gas Chromatography

Gas chromatography with flame ionization detection (GC-FID) was used to determine the fatty acid side chains in the purified MEL after derivatization to the corresponding methyl esters. First, 10 mg of MEL were mixed with 1.5 ml of 5% acetyl chloride in methanol and vortexed for 1 min, before the samples were incubated at 100°C for 1.5 h and left to cool. Then, 1.5 ml of water and 2 ml of hexane containing an internal standard (C_15_-ME) were added and the samples were vortexed for 1 min. The phases were separated by centrifugation (5000 rpm, 10 min) and the hexane phase containing the methyl esters transferred into GC vials. GC analysis was performed on an Agilent 7890A GC-FID system with a SPB-PUFA capillary column (30 m × 0.32 mm, d_f_ = 0.2 μm; Sigma-Aldrich, USA). Helium at 50 ml/min was used as carrier gas and the temperature gradient ranged from 60°C to 220°C. Injection volume was 1 μl. A Supelco 37 Component FAME Mix (Sigma-Aldrich, USA), containing linear saturated and unsaturated fatty acids, was used as the reference standard.

### High-Performance Thin Layer Chromatography With Matrix-Assisted Laser Desorption Ionization Time-of-Flight Mass Spectrometry

High performance thin layer chromatography (HPTLC) coupled to matrix-assisted laser desorption ionization time-of-flight mass spectrometry (MALDI-TOF-MS) was performed as described previously (Beck et al., 2019). Normal phase HPTLC plates (HPTLC silica 60, 5 × 7.5 cm, Merck, Germany) and a solvent system consisting of chloroform and methanol (20:3 v/v) were used for separating the chromatographically purified MELs by polarity. Sample application was done using an ATS4 automatic TLC sampler (CAMAG, Muttenz, Switzerland). After development, the plates were coated with a matrix solution consisting of 200 g/l dihydroxybenzoic acid, 1.15 g/l ammonium dihydrogen phosphate, and 0.1% trifluoroacetic acid in acetonitrile/water (9:1, v/v) (Günther, 2014). Diluted polypropylene glycol 725 (Sigma-Aldrich, USA) was spotted into the corners of the HPTLC plate and used as external standard to verify the correct mass calibration of the MS system. MALDI-TOF analysis was performed on a Bruker Ultraflex II TOF/TOF controlled by flexControl software (Bruker Daltonics, USA). The plates were scanned over the entire running distance with an interval of 0.6 mm and each spectrum was summed from five spots at 200 laser shots each (m/z range 200–2000 Da). Data evaluation was performed with Bruker flexAnalysis and TLC MALDI software (Bruker Daltonics, USA). For visualization purposes, a duplicate of the same HPTLC plate was created and stained with a solution of acetic acid/*p*-anisaldehyde/sulfuric acid (97:1:2 v/v/v).

### High-Performance Liquid Chromatography With Electrospray Ionization Mass Spectrometry

Analysis of the pure MEL samples by high-performance liquid chromatography with electrospray ionization mass spectrometry (HPLC-ESI-MS) was performed on a PE Series 200 HPLC system equipped with an Applied Biosystems API 150 mass spectrometer. Data collection was done using Analyst 1.3 software. A Merck Select B (250×4 mm, 5 mm) column was used as stationary phase. Eluent A: 5 mM ammonium formate + 0.1% formic acid and eluent B: acetonitrile/methanol = 1:1, 5 mM ammonium formate + 0.1% formic acid (pH 3) were used as mobile phase. Elution was performed at 0.9 ml/min with a gradient ranging from 15% B at 0 min to 100% B at 30 min, before holding 100% B until 40 min. Samples were prepared as 30 mg/ml solutions in DMSO and injected with 1 μl. Detection was performed with a UV diode array detector (200–800 nm), evaporative light scattering detector (Sedex 75, parallel split), and by (+/−)-ESI- MS in fast-switching mode. Data analysis was performed with Analyst 1.3 (Sciex) and MNova 14.1 (Mestrelab, Spain).

### NMR Spectroscopy

For NMR spectroscopy, approximately 5 mg of pure MEL compounds, which were obtained by preparative HPLC, were dissolved in 0.6 ml methanol-d_4_. H-NMR, HSQC, HMBC, and HH-COSY spectra were measured on a Bruker AVANCE NEO 400 spectrometer (400 MHz) and the spectra were processed with Topspin 4.0 software (Bruker).

## Results

### Production and Purification of MEL From Castor Oil

The production process to obtain MELs from castor oil with the seven investigated organisms was initiated by feeding 8% castor oil to the culture after the cells had consumed glucose as their primary growth substrate and entered into stationary phase (t = 72 h). Cultivation was then maintained for a total of 504 h to achieve a sufficient amount of MEL (around 1–5 g/l) for the successive structural analysis.

HPTLC analysis of the culture broth extracts during cultivation with castor oil showed a different composition when compared to those from rapeseed oil (supplementary Fig. S1). Whereas with rapeseed oil, there was only one peak for triacylglycerol and one for the free fatty acids; multiple peaks were obtained for the hydrolysis products from castor oil. The MEL pattern, which is usually comprised of MEL-A, -B, -C, and minor amounts of MEL-D along with some mannosylmannitol lipid (MML) congeners, was also altered with castor oil. We could observe multiple peaks with similar retention time to conventional MELs, which however did not correspond to previously known structures.

After the cultivation was terminated, the entire broth was extracted and the crude extract was subsequently purified by flash column chromatography. The production and purification process was the same as reported previously for MEL from rapeseed, soybean, or olive oil (Beck et al., 2019). The purified MELs were then used for detailed structure analysis.

### Fatty Acid Profiles of the MELs From Castor Oil Compared to Rapeseed Oil

Gas chromatographic analysis was used to determine the fatty acids incorporated into the side chains of the purified MELs. An overview of fatty acids in the respective MELs from castor oil, compared to those from rapeseed oil (previously published in Beck et al., 2019), are shown in Table 1 for all seven investigated species. It was interesting to see that there were two novel fatty acid peaks, which had not been observed before with conventional vegetable oils like rapeseed, soybean, or olive oil. As they were also not present in the standard fatty acid methyl ester (FAME) mix that was used for calibration and verification, it was obvious that these were unusual fatty acids commonly not found in natural products. The retention time of those two fatty acids was between those of common fatty acids like C_16_ or C_18_ and ricinoleic acid (C_18:1-OH_). Thus, our hypothesis was that these were novel hydroxylated fatty acids with a chain length below 18 carbon atoms, possibly 10-hydroxy-7-cis-hexadecenoic acid (C_16:1-OH_) and 8-hydroxy-5-cis-tetradecenoic acid (C_14:1-OH_). This was later verified by LC-MS analysis and NMR. The first of the two novel fatty acid peaks, which was consequently attributed to C_14:1-OH_, was present in castor oil MELs from all seven species; while the latter, C_16:1-OH_, was only found in MELs from *M. aphidis, U. siamensis*, and *U. shanxiensis;* and in lower concentrations. The latter three organisms generally had the highest shares of hydroxylated fatty acids with 16.1%, 22.3%, and 31.6%, respectively. In the other four species, those shares were below 7% of total fatty acids. Thus, the occurrence of the C_16:1-OH_ fatty acid might be related to the higher concentration of hydroxylated fatty acids in *M. aphidis, U. siamensis*, and *U. shanxiensis.*

**Table 1.** Gas Chromatographic Analysis of Fatty Acid Residues Found in MEL Produced From Castor Oil or Rapeseed Oil. Composition of Substrate Oils Is Also Shown for Comparison. Analysis of MELs From Castor Oil Shows Two Additional Peaks That Can Be Linked to Hydroxylated Fatty Acids With a Chain Length of 14 Carbon Atoms (C_14:1-OH_) and 16 Carbon Atoms (C_16:1-OH_)

	Fatty acid methyl esters (area %)															
	*Moesziomyces aphidis*		*Ustilago siamensis*		*Ustilago shanxiensis*		*Pseudozyma hubeiensis*		*Sporisorium graminicola*		*Pseudozyma tsukubaensis*		*Moesziomyces parantarcticus*		Substrate oil	
Fatty acid	CAS	RSO	CAS	RSO	CAS	RSO	CAS	RSO	CAS	RSO	CAS	RSO	CAS	RSO	CAS	RSO
**C4:0-ME**	n.a.	n.a.	n.a.	n.a.	n.a.	n.a.	n.a.	n.a.	n.a.	n.a.	n.a.	n.a.	n.a.	n.a.	n.a.	n.a.
**C6:0-ME**	1.2	**-**	**-**	**-**	5.2	**-**	**34.0**	**25.7**	**29.9**	**9.6**	**-**	1.2	**11.5**	**-**	**-**	**-**
*	**-**	**-**	**-**	**-**	**-**	**-**	**-**	2.9	**-**	**-**	**-**	**-**	**-**	**-**	**-**	**-**
**C8:0-ME**	**40.3**	**12.7**	**-**	**-**	3.7	**-**	**-**	2.5	**39.6**	**30.3**	**64.1**	**30.2**	**46.7**	**25.2**	**-**	**-**
*	**-**	4.5	**-**	**-**	**-**	**-**	**-**	**-**	**-**	3.3	**-**	8.7	**-**	**9.4**	**-**	**-**
*	**-**	**-**	**-**	**-**	**-**	**-**	**-**	3.9	**-**	**-**	**-**	**-**	**-**	**-**	**-**	**-**
**C10:0-ME**	4.2	**51.3**	**-**	**-**	**-**	**-**	**18.1**	**30.2**	**-**	3.6	**23.5**	**29.1**	**20.4**	**48.4**	**-**	**-**
*	**-**	**14.2**	**-**	**-**	**-**	**-**	**-**	**-**	**-**	**-**	**-**	2.9	1.6	3.4	**-**	**-**
*	2.2	1.9	**-**	**-**	**-**	**-**	1.5	**-**	**-**	**-**	5.8	1.6	2.2	1.5	**-**	**-**
*	**-**	8.5	**-**	**-**	**-**	**-**	**-**	**-**	**-**	**-**	**-**	4.1	**-**	3.8	**-**	**-**
*	1.2	**-**	**-**	**-**	**-**	**-**	1.0	**-**	**-**	**-**	**-**	**-**	**-**	**-**	**-**	**-**
**C12:0-ME**	1.9	3.9	**-**	**-**	1.1	**-**	**24.6**	**17.5**	5.4	**9.6**	3.3	7.3	7.3	6.6	**-**	**-**
*	**-**	**-**	**-**	**-**	**-**	**-**	**-**	1.8	**-**	**-**	**-**	5.9	**-**	**-**	**-**	**-**
*	**-**	**-**	**-**	**-**	**-**	**-**	**-**	1.4	**-**	**-**	**-**	2.3	**-**	**-**	**-**	**-**
*	**-**	**-**	**-**	**-**	**-**	**-**	1.4	**-**	**-**	**-**	**-**	**-**	**-**	**-**	**-**	**-**
**C14:0-ME**	2.3	**-**	6.5	3.6	2.7	2.5	4.4	**-**	**9.2**	**11.4**	**-**	**-**	3.3	**-**	**-**	**-**
*	**-**	**-**	**-**	**19.6**	1.6	**13.4**	**-**	**-**	1.5	**12.4**	**-**	3.6	**-**	**-**	**-**	**-**
*	**-**	**-**	**-**	**-**	**-**	**-**	**-**	**-**	1.2	5.5	**-**	**-**	**-**	**-**	**-**	**-**
*	**-**	1.3	**-**	**13.7**	**-**	7.0	**-**	**-**	**-**	3.4	**-**	**-**	**-**	**-**	**-**	**-**
*	**-**	**-**	**-**	**-**	1.2	**-**	**-**	1.6	**-**	**-**	**-**	**-**	**-**	**-**	**-**	**-**
*	**-**	1.7	**-**	**-**	**-**	**-**	**-**	**-**	**-**	**-**	**-**	1.8	**-**	**-**	**-**	**-**
**C16:0-ME**	**19.9**	**-**	**55.3**	**24.2**	**29.1**	**23.1**	4.7	**-**	4.8	**-**	**-**	**-**	**-**	**-**	**-**	5.4
*	3.9	**-**	4.9	**29.8**	7.6	**38.8**	1.0	**-**	1.0	3.9	**-**	**-**	**-**	**-**	**-**	**-**
**C16:1n7-ME**	2.9	**-**	**-**	3.9	5.7	2.9	**-**	**-**	**-**	1.3	**-**	**-**	**-**	**-**	**-**	**-**
*	**-**	**-**	**-**	2.8	**-**	2.8	4.4	9.0	**-**	2.6	**-**	**-**	**-**	**-**	**-**	**-**
*	**-**	**-**	**-**	**-**	**-**	**-**	1.5	3.4	**-**	3.3	**-**	**-**	**-**	**-**	**-**	**-**
**C18:0-ME**	**-**	**-**	**-**	**-**	**-**	**-**	**-**	**-**	**-**	**-**	**-**	**-**	**-**	**-**	1.3	**-**
**C18:1n9c-ME**	2.3	**-**	8.0	2.5	4.2	4.1	**-**	**-**	1.2	**-**	**-**	1.5	**-**	1.9	2.9	**62.0**
*	**-**	**-**	**-**	**-**	**-**	**-**	**-**	**-**	**-**	**-**	**-**	**-**	**-**	**-**	0.5	3.5
**C18:2n6c-ME**	1.5	**-**	2.9	**-**	2.2	1.6	**-**	**-**	**-**	**-**	**-**	**-**	**-**	**-**	4.4	**19.9**
**C18:3n3-ME**	**-**	**-**	**-**	**-**	**-**	2.1	**-**	**-**	**-**	**-**	**-**	**-**	**-**	**-**	**-**	8.0
**C14:1-OH-ME**	**12.2**	**-**	**16.8**	**-**	**21.9**	**-**	1.8	**-**	4.1	**-**	3.2	**-**	7.0	**-**	**-**	**-**
*	**-**	**-**	**-**	**-**	**-**	1.6	**-**	**-**	**-**	**-**	**-**	**-**	**-**	**-**	**-**	**-**
**C20:1n9-ME**	**-**	**-**	**-**	**-**	**-**	**-**	**-**	**-**	**-**	**-**	**-**	**-**	**-**	**-**	**-**	1.2
*	**-**	**-**	**-**	**-**	1.0	**-**	**-**	**-**	**-**	**-**	**-**	**-**	**-**	**-**	**-**	**-**
**C16:1-OH-ME**	2.1	**-**	5.5	**-**	2.9	**-**	**-**	**-**	**-**	**-**	**-**	**-**	**-**	**-**	**-**	**-**
*	**-**	**-**	**-**	**-**	3.1	**-**	**-**	**-**	**-**	**-**	**-**	**-**	**-**	**-**	**-**	**-**
**C18:1n9-OH-ME (Ricinoleic acid)**	1.8	**-**	**-**	**-**	6.8	**-**	1.5	**-**	2.1	**-**	**-**	**-**	**-**	**-**	**88.0**	**-**
**Saturated acids**	69.8	67.9	61.8	27.8	41.8	25.6	85.8	75.9	88.9	64.5	90.9	67.8	89.2	80.2	1.3	5.4
**Unsaturated acids**	30.1	32.1	38.1	72.3	58.2	74.3	14.1	24	11.1	35.7	9	32.4	10.8	20	98.7	94.6
**Hydroxylated acids**	16.1	0	22.3	0	31.6	0	3.3	0	6.2	0	3.2	0	7	0	88	0

*Note.* Results are expressed in relative area percent of fatty acid methyl esters after derivatization. The most abundant fatty acids (rel. area percentage > 9%) are highlighted in bold.

CAS = castor oil; RSO = rapeseed oil;

n.a. = C4:0-ME could not be quantified due to interference with derivatization agents, *mono- or poly-unsaturated fatty acids. Unsaturated fatty acids were eluted after the respective saturated fatty acids.

Besides observation of completely novel fatty acids, the chain length and degree of saturation of conventional fatty acids was also slightly altered for some species when compared with the MELs from rapeseed oil. In *M. aphidis* MEL from castor oil, we found mainly C_8:0_ (40.3%), C_16:0_ (19.9%) and the already mentioned C_14:1-OH_ (12.2%) fatty acids. In contrast, C_10:0_ fatty acids, which are quite abundant in *M. aphidis* MEL from rapeseed oil (51.3%), were only included in minor concentrations with castor oil (4.2%). It was hence obvious that castor oil had a strong influence on the MEL structures produced by *M. aphidis* and that this MEL mixture needed further investigation to deduce the exact combination of fatty acids.

In *U. siamensis* and *U. shanxiensis* MEL from castor oil, C_16:0_ and C_14:1-OH_ were the most abundant fatty acids. Overall, the amount of conventional C_14_ acids and the amount of unsaturated fatty acids were decreased compared to rapeseed oil. For example, the share of unsaturated C_16_ fatty acids (sum of fatty acids between C_16:0_ and C_18:0_) decreased from 36.5% in rapeseed oil MEL to only 4.9% in castor oil MEL for *U. siamensis* and from 44.5% to 13.3% for *U. shanxiensis*, while the amount of C_16:0_ was increased respectively. This seemed to be contra-intuitive, since castor oil does not contain saturated fatty acids at all. A possible explanation would be the *de-novo*-synthesis of fatty acids from acetyl-CoA, which will be discussed later.

For the other four species—*P. hubeiensis, P. tsukubaensis, S. graminicola*, and *M. parantarcticus*—the fatty acid pattern was similar between castor and rapeseed oil. However, the total MEL concentration with castor oil was much lower and they only had a low share of hydroxylated fatty acids in their MEL. It was therefore concluded that those strains are not able to produce large amounts of MEL from castor oil under the used conditions.

### Structure Elucidation by Mass Spectrometry

After characterization of the fatty acid profiles, the next step was to investigate the novel MEL structures from castor oil, that is, their molecular weight, acetylation pattern, and combination of fatty acid residues. A special focus will be put on the novel MELs from *M. aphidis, U. siamensis*, and *U. shanxiensis*, as they were the best producers of those structures.

#### HPTLC and MALDI-TOF-MS Analysis

**Fig. 2.**
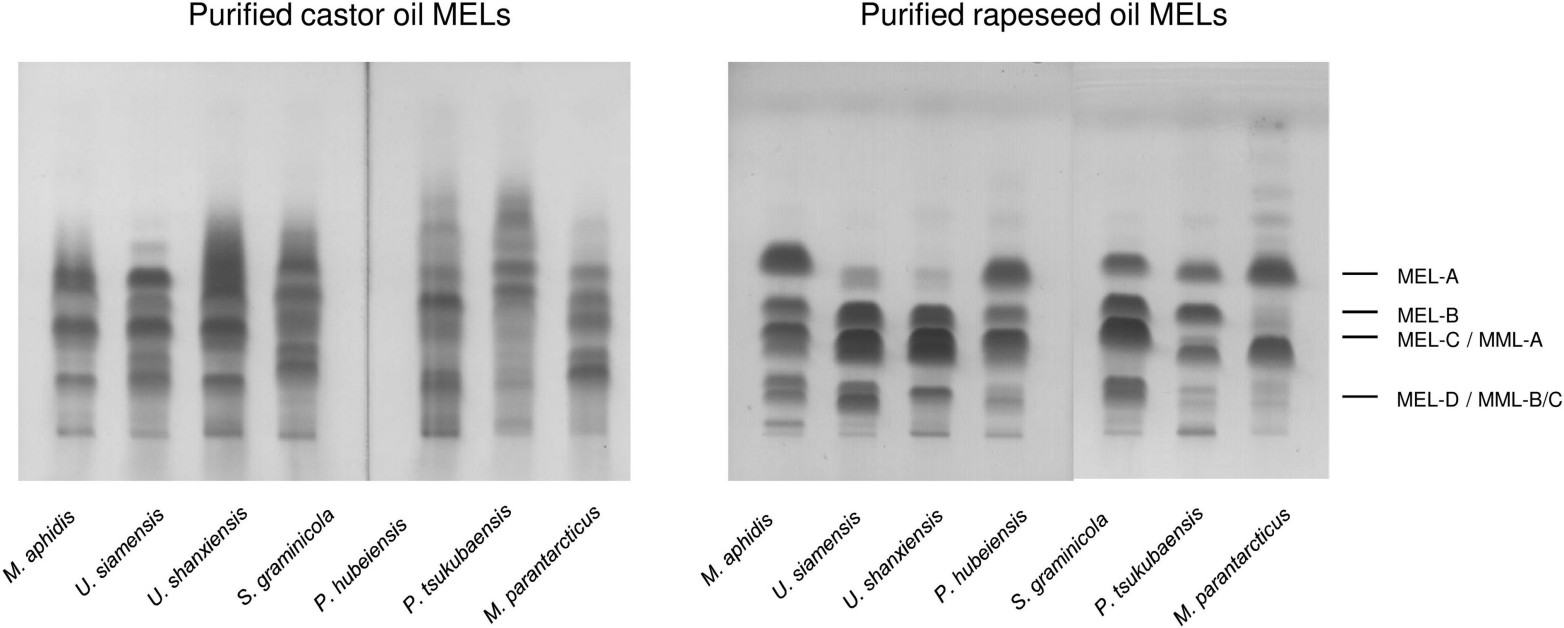
HPTLC analysis of chromatographically purified MEL fractions from cultivations with castor oil and rapeseed oil for the seven investigated *Ustilaginaceae* species. MELs from castor oil show a more complex pattern than from rapeseed oil and could only be resolved by mass spectrometry

Previously, we had reported a method combining HPTLC and MALDI-TOF mass spectrometry to deduce the structures of MEL from rapeseed, soybean, and olive oil (Beck et al., 2019). To elucidate the chemical structure of the novel MELs from castor oil, the same method was used first. After HPTLC separation of the chromatographically purified MELs from castor oil, there were more peaks present than just the conventional MEL-A, -B, -C, and –D congeners obtained from rapeseed oil (Fig. 2). This had already been observed during cultivation. Using MALDI-TOF-MS, we determined the respective molecular masses [M + Na]^+^ within the different TLC peaks. The detailed procedure has been published before (Beck et al., 2019) and is shown exemplarily in supplementary Fig. S2. A summary of the detected ions for all seven microorganisms is presented in Table 2.

**Table 2.** Detected Ions [M + Na]^+^ From MALDI-TOF-MS Analysis of Castor Oil MELs From All Seven *Ustilaginaceae* Species, Sorted by Their Retention Behavior on HPTLC (Influenced by Acetylation Degree). Classification Into the Different Types (MELs, MMLs, Hydroxy-Di-MELs, and Hydroxy-Tri-MELs) Was Done Based on Theoretical Mass Calculations and Comparison With Measured Ions. Masses Highlighted in Bold Were the Most Abundant Ions of Each MEL Type

MEL type/species	*Moesziomyces aphidis*	*Ustilago siamensis*	*Ustilago shanxiensis*	*Pseudozyma hubeiensis*	*Sporisorium graminicola*	*Pseudozyma tsukubaensis*	*Moesziomyces parantarcticus*
**MEL-A**	699.9; 671.8; 669.8; **643.9;** 615.8	N/A	N/A	699.2; **671.1;** 643.1	727.5; 699.5; 671.5	**671.6**; 669.6; 643.5	727.5; 699.6; **671.5**; 669.5; 643.5
**Hydroxy-MEL-A**	713.4	N/A	N/A	657.2	N/A	N/A	N/A
**Hydroxy-tri-MEL-A**	923	N/A	923	N/A	N/A	N/A	N/A
**MEL-B**	657.5; 629.4; 627.4; **601.3**	**657.9**; 655.8; 629.9	**657.2**; 655.5; 629.3	657.2; 629.2; 601.1	685.8; **657.7;** **629.7**	713.5; 687.5; 657.5; 655.5; 627.5	657.5; 629.5; 601.5
**MEL-C**	657.5; 629.4; 627.4; **601.3**	683.5; **657.9**; 655.8; 629.9; 627.5	**657.2**; 655.5; 629.3; 627.5	657.2; **629.2;** 601.1	685.7; **657.7;** 629.7; 601.6	N/A	657.5; 629.5; 601.5
**Hydroxy-MEL-B/C**	671.5; **643.4**	671.5; **643.5**	671.5; **643.5**	671.6; 643.5	699.5; 671.5; 643.5	699.5; **697.5;** 671.5; 643.5	N/A
**Hydroxy-tri-MEL-B/C**	935; 909; **907**; 905	935; 909; **907**881	909; **907**; 905	N/A	N/A	N/A	N/A
**MML-A**	N/A	N/A	N/A	731.5; 701.4	N/A	731.7; 703.7	**731.6;** 703.6;
**MEL-D**	615.4	615.5; 613.5	615.5	N/A	643.5; 641.5	N/A	N/A
**MML-B/C**	N/A	717.2;	N/A	N/A	717.9; 689.7	N/A	N/A
**Hydroxy-MEL-D**	601.2	**601.1**	**601.1**	629.4; 601.4	657;5; 629.5; 627.5	N/A	657.5; 629.9
**Hydroxy-tri-MEL-D**	865	893; **865**	N/A	N/A	N/A	N/A	N/A

In general, multiple ions corresponding to conventional MEL-A to MEL-D variants were observed, but also others with slightly lower retention and a mass difference of +14 or +16 Da, characteristic for an additional oxygen atom and one or no double bond (+/−2 H-atoms). Based on a theoretical calculation of molecular masses, those ions correlate to MEL structures containing a hydroxylated fatty acid with one unsaturation (i.e., double bond). For example, ions of m/z 643.5 and 671.5 Da [M + Na]^+^ were detected for all seven species with castor oil, which correspond to hydroxylated MEL-B/C with an overall fatty acid chain length of 18 or 20 carbon atoms, with one double bond and one hydroxyl group. Combined with the results from GC analysis, structures of MEL-B/C-C_4_-C_14:1-OH_ and MEL-B/C-C_4_-C_16:1-OH_ were thus proposed for *U. siamensis* and *M. aphidis* for example. The related conventional MEL-B/C variants with nonhydroxylated saturated fatty acids were detected at m/z 601.5, 629.5, and 657.5 Da [M + Na]^+^, respectively (e.g., MEL-B/C-C_4_-C_14:0_ and MEL-B/C-C_4_-C_16:0_ for *U. siamensis*). For *M. aphidis*, ions of m/z 713.5 Da [M + Na]^+^ with a similar retention to MEL-A were observed that would correspond to novel hydroxylated MEL-A with a total fatty acid chain length of 20 carbon atoms, one double bond and one hydroxyl group, respectively; for example, MEL-A-C_6_-C_14:1-OH_ or MEL-A-C_4_-C_16:1-OH_. Ions corresponding to hydroxylated MEL-D were also detected at m/z 601.2 and 629.4 Da [M + Na]^+^, which agrees with structures of MEL-D-C_4_-C_14:1-OH_ and MEL-D-C_4_-C_16:1-OH_.

Besides these novel di-acylated congeners bearing a hydroxy fatty acid, we could also detect ions with larger m/z values of, for example, m/z 935.8, 923.8, 909.8, 907.8, 905.8, 893.8, or 865.8 Da [M + Na]^+^ for *M. aphidis, U. siamensis*, and *U. shanxiensis*. These structures showed similar retention factors to conventional MELs and were present in low quantities. Such comparably high masses above 850 Da have been reported for MEL structures, where an additional third fatty acid is esterified to the MEL molecule (tri-MELs) (Fukuoka et al., 2007a; Goossens et al., 2016; Morita et al., 2008). As these authors have shown, the third fatty acid can be bound to the terminal hydroxyl group of the erythritol moiety, resulting from lipase-catalyzed reaction with residual substrate fatty acids in the broth. The additional acylation of the erythritol moiety in these tri-MELs made them much more hydrophobic than conventional di-acylated MELs (Goossens et al., 2016). In our case however, the third fatty acid could also be bound to the additional hydroxyl group found in the side chains of the novel hydroxylated MELs. This would be consistent with the retention factors of our novel MEL structures, which were similar to those of conventional di-acylated MELs. As ‘classical’ tri-MELs with a third fatty acid at the erythritol moiety would be much more hydrophobic and therefore show a different migration behavior, the ions that we detected from castor oil had to be novel hydroxylated tri-acylated MEL structures. For example, ions of m/z 907.8 Da [M + Na]^+^, which were the most abundant of those high-molecular mass ions in MELs from *M. aphidis, U. siamensis*, and *U. shanxiensis* with castor oil, could be the result of a novel MEL-B/C-C_4_-C_14:1-OH_ esterified with an additional C_18:1_ fatty acid, thus yielding tri-MEL-B/C-C_4_-C_14:1-OH_-C_18:1_. Accordingly, the ion with m/z 935.8 Da [M + Na]^+^, which has mass difference of one C2 unit (+28 Da), can be related to tri-MEL-B/C-C_4_-C_16:1-OH_-C_18:1_. Ions with m/z 923.8 Da [M + Na]^+^, which were, for example, detected in castor oil MEL from *M. aphidis*, could be novel tri-MEL-A-C_4_-C_14:1-OH_-C_16:0_. Ultimately, ions of m/z 865.8 and 893.8 Da [M + Na]^+^ would correspond to tri-MEL-D-C_4_-C_14:1-OH_-C_18:1_ and tri-MEL-D-C_4_-C_16:1-OH_-C_18:1_.

#### HPLC-ESI-MS With Fragmentation Analysis

To verify the several novel hydroxy MEL structures that were proposed based on MALDI-TOF-MS analysis and to deduce the combination of their fatty acid residues, reversed-phase HPLC with gradient elution and electrospray ionization (ESI)-MS detection was employed. The major benefit of using ESI-MS is the possibility to analyze multiple fragment ions, yielding a more detailed understanding of the molecular structure than just the parental ions generated in MALDI-TOF-MS. In reversed-phase HPLC, which is commonly used along with ESI-MS, the MEL mixtures are moreover separated according to their fatty acid pattern. This is resulting in a separate peak for each fatty acid combination (see also Onghena et al., 2011); while on TLC with a normal-phase system, the mixtures are separated mainly according to acetylation degree (MEL-A to MEL-D). Thus, a different peak pattern could be obtained by changing the chromatographic separation principle. This allowed us to discriminate between MELs with conventional fatty acids and those containing hydroxylated fatty acids.

**Fig. 3.**
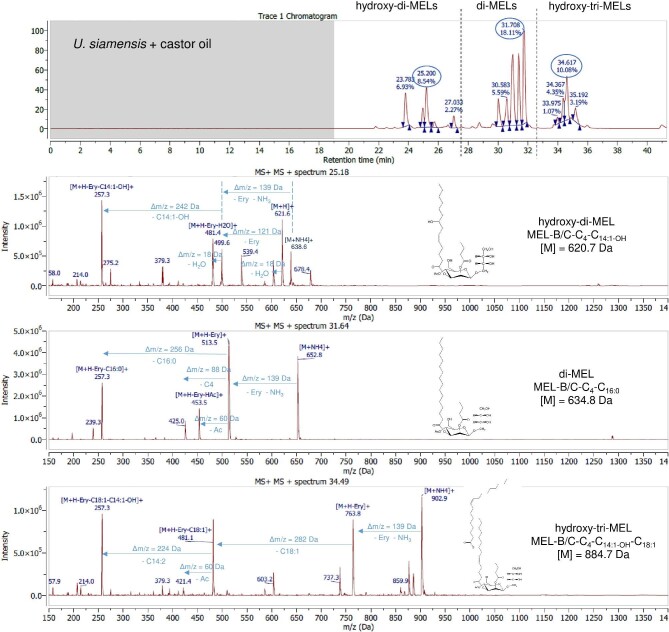
Chromatogram and selected mass spectra from positive ion mode ESI(+)-MS fragmentation analysis of three representative HPLC peaks at t_R_ = 25.2 min (hydroxy-di-MEL), t_R_ = 31.6 min (di-MEL), and t_R_ = 34.5 min (hydroxy-tri-MEL) for *Ustilago siamensis* MEL with castor oil.

**Fig. 4.**
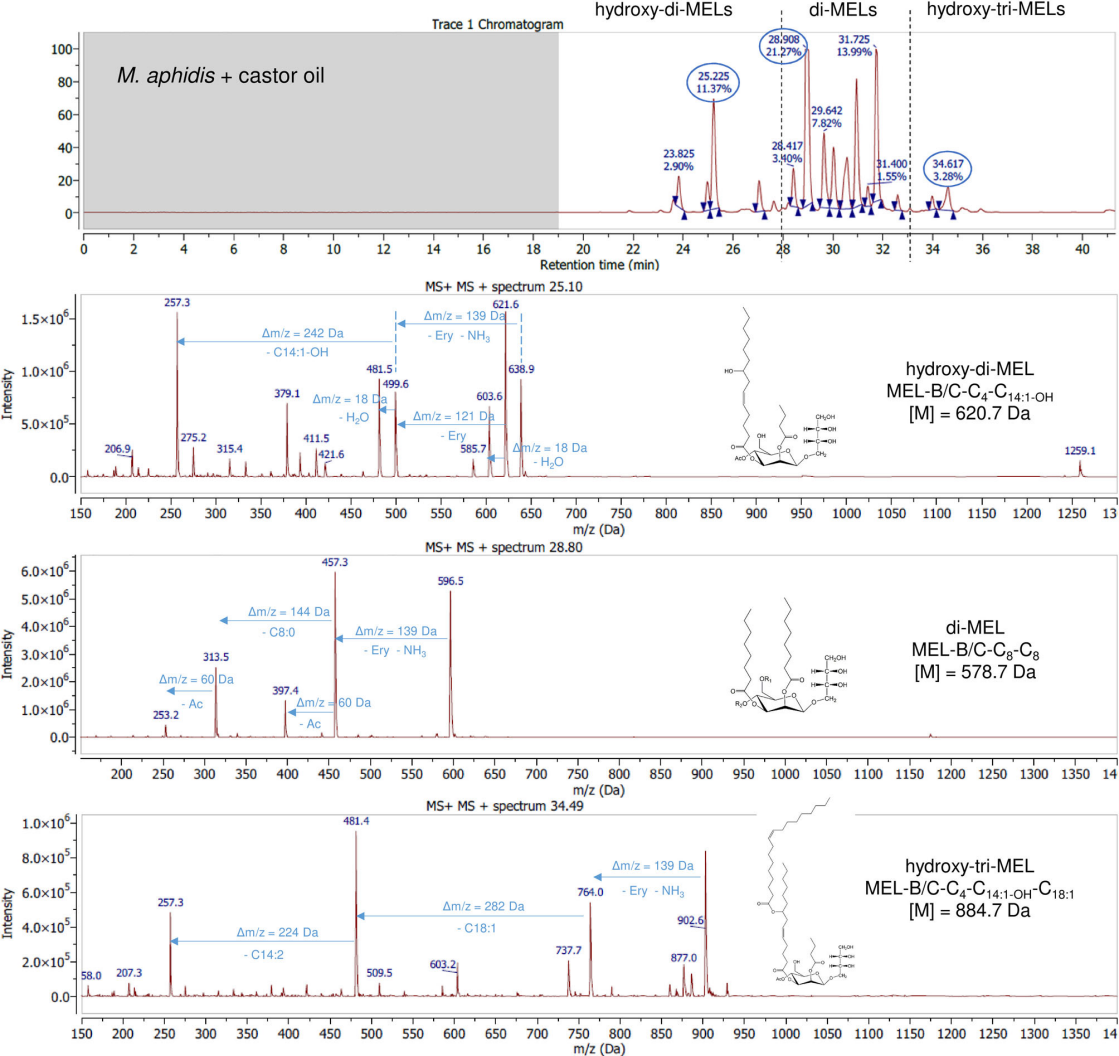
Chromatogram and selected mass spectra from positive ion mode ESI(+)-MS fragmentation analysis of three representative HPLC peaks at t_R_ = 25.1 min (hydroxy-di-MEL), t_R_ = 28.8 min (di-MEL), and t_R_ = 34.5 min (hydroxy- tri-MEL) for *Moesziomyces aphidis* MEL with castor oil.

Resulting LC chromatograms of all MELs from castor oil and rapeseed oil are given in the supplementary files (Supplementary Figs. S3–S8). It was again observed that the composition of MELs from castor oil is much more diverse than from rapeseed oil, as already seen during MALDI-TOF-MS analysis. While only conventional di-acylated MELs were formed with rapeseed oil (t_R_ = 28–33 min, di-MELs), there were many additional peaks in the chromatograms of castor oil MELs. Those could be linked to either di-acylated MELs with a hydroxy fatty acid (t_R_ = 22–28 min, hydroxy-di-MELs) or tri-acylated MELs with a hydroxy fatty acid (t_R_ = 33–37 min, hydroxy-tri-MELs), which are grouped accordingly in the chromatograms. In Fig. 3 and Fig. 4, a detailed LC-MS analysis of *U. siamensis* and *M. aphidis* MEL from castor oil, with representative mass spectra of the highest peaks from each group, is presented. Based on those two examples, the fragmentation principles that were used for detailed structural analysis of individual MEL congeners from each group are explained in the following. Still, the general procedure was the same for all seven species.

For conventional di-acylated MELs (di-MELs) with a retention time between t_R_ = 28 min and t_R_ = 33 min, two main ions with high intensity were observed. The first was the [M + NH_4_]^+^ adduct ion, which can be explained by the presence of ammonium salt in the mobile phase. The second was a characteristic ion with Δm/z = 139 Da mass difference, which could be linked to a neutral loss of the erythritol moiety plus ammonium adduct [M + H-erythritol]^+^. Cleavage of the glycosidic bond is a common fragmentation pathway for glycosylated biomolecules in ESI-MS and has been observed before for MELs (Onghena et al., 2011). Additional fragment ions with lower abundance were found at [M + H-erythritol-HAc]^+^ and [M + H-erythritol-fatty acid]^+^, resulting from the cleavage of ester bonds between the respective fatty acid/acetyl group and the mannose moiety. As an example, the di-acylated MEL-B/C-C_4_-C_16:0_ from *U. siamensis* with castor oil (t_R_ = 31.6 min, M = 634.8 Da) led to ions of m/z 652.8 Da [M + NH_4_]^+^, 513.5 Da [M + H-erythritol]^+^, 453.5 Da [M + H-erythritol-AcOH]^+^, 425.5 Da [M + H-erythritol-C_4_]^+^, and 257.3 Da [M + H-erythritol-C_16:0_]^+^. Results for conventional di-acylated MEL from *M. aphidis* with castor oil were quite surprising. *M. aphidis* MEL from rapeseed oil contains high amounts of MEL-A along with lesser amounts of MEL-B and -C. The most common fatty acid combinations with rapeseed oil are C_8_–C_10_ or C_10_–C_10_ (Beck et al., 2019; Rau et al., 2005). In contrast, the *M. aphidis* di-MELs from castor oil, found between t_R_ = 28 min and t_R_ = 33 min, showed a different fragmentation pattern. The largest peak at t_R_ = 28.8 min showed ions of m/z 596.8 Da [M + NH_4_]^+^, 457.3 Da [M + H-erythritol]^+^, 397.4 Da [M + H-erythritol-AcOH]^+^, and 313.5 Da [M + H-erythritol-C_8_]^+^. Based on this fragmentation pattern, a structure of MEL-B/C-C_8_-C_8_ (M = 578.7 Da) was deduced as the main congener. Combinations of C_10_-C_10_ were not observed in *M. aphidis* MEL from castor oil. While this is very different to the rapeseed oil MELs, it confirms the observations that were already made when comparing the GC analysis of *M. aphidis* MELs. A large amount of C_8_ fatty acids and low amounts of C_10_ were observed with castor oil MEL in GC, which is in accordance with the results of LC-MS analysis. It can thus be stated that castor oil as substrate had a strong influence on the fatty acid composition of MEL from *M. aphidis*.

The novel hydroxylated di-acylated MELs (hydroxy-di-MELs) found between t_R_ = 22 min and t_R_ = 28 min showed a complex fragmentation pattern in LC-MS. As such, they could be clearly distinguished from conventional di-MELs. For the novel hydroxylated MELs, the molecular ion peak [M + H]^+^ was detected with highest intensity, along with the ammonium adduct ion [M + NH_4_]^+^. The free OH-group in the carbon chain of the fatty acid could also lead to a dehydration reaction, resulting in a [M + H-H_2_O]^+^ ion with Δm/z = −18 Da and lower abundance. Neutral loss of erythritol also occurred, leading to [M + H-erythritol]^+^ and [M + H-erythritol-H_2_O]^+^. Similar to conventional di-MELs, an additional neutral loss of the hydroxy fatty acid ultimately led to an [M + H-erythritol-fatty acid]^+^ ion, which was characteristic for a neutral loss of either C_14:1-OH_ or C_16:1-OH_ fatty acids (Δm/z = −242 or 270 Da). For example, MEL-B/C-C_4_-C_14:1-OH_ from *U. siamensis* (t_R_ = 25.2 min, M = 620.7 Da) showed fragment ions of m/z 638.8 Da [M + NH_4_]^+^, 621.7 Da [M + H]^+^, 499.6 Da [M + H-erythritol]^+^, 481.4 Da [M + H-erythritol-H_2_O]^+^, and 257.3 Da [M + H-erythritol-C_14:1-OH_]^+^. Similar structures were obtained for *M. aphidis*.

The fragmentation pattern of the proposed tri-acylated MELs with one hydroxy fatty acid (hydroxy-tri-MELs), found between t_R_ = 33 min and t_R_ = 37 min, confirmed their unique structural composition. Starting from the ammonium adduct ion [M + NH_4_]^+^, first a neutral loss of erythritol and ammonium was observed, which led to [M + H-erythritol]^+^ (Δm/z = 139 Da). Additionally, neutral loss of a long-chain fatty acid and/or a hydroxy fatty acid was detected. For example, the molecule at t_R_ = 34.5 min in *U. siamensis* MEL from castor oil showed characteristic ions of m/z 902.9 Da [M + NH_4_]^+^, 763.8 Da [M + H-erythritol]^+^, 481.1 Da [M + H-erythritol-C_18:1_]^+^, and 257.3 Da [M + H-erythritol-C_18:1_-C_14:1-OH_]^+^. Thus, the structure of this molecule was identified as MEL-B/C-C_4_-C_14:1-OH_-C_18:1_ (M = 884.7 Da), where the third C_18:1_ fatty acid must be esterified to the hydroxyl group of the C_14:1-OH_ acid. This was later confirmed by NMR. Again, similar structures were found for *M. aphidis*.

As shown for *U. siamensis* and *M. aphidis* MELs exemplarily, the mass spectrometric fragmentation pattern could be used like a fingerprint to deduce the chemical structure of each molecule in the MEL mixtures. The order of elution in reversed-phase chromatography was as follows: Fatty acid side chains had the strongest influence on elution behavior; therefore, MEL structures containing hydroxylated fatty acids (hydroxy-di-MELs) were eluted before classical di-acylated MELs (di-MELs). Tri-acylated MELs (Hydroxy-tri-MELs) were eluted last. In addition, the chain length of fatty acids had a stronger influence than the acetylation pattern, so that, for example, a MEL-A with a combined fatty acid length of C18 was eluted before a MEL-B/C with a combined fatty acid length of C20, respectively. MEL structures with higher degree of unsaturated fatty acids were eluted earlier than those with equal chain length but less double bonds.

Based on this detailed HPLC-MS analysis, all chemical structures within the novel MELs from castor oil that were postulated based on GC and MALDI-TOF analysis could be confirmed. A detailed summary for the promising producer organisms *M. aphidis and U. siamensis* is shown in Tables 3 and 4. A summary for *U. shanxiensis*, which was also producing high shares of hydroxyl MEL congeners similar to *U. siamensis*, can be found in the Supplementary Table S9. *M. aphidis* MEL from castor oil had a lower share of hydroxylated structures than the other two organisms. Around 75% of the mixture were still conventional di-acylated MELs, while roughly 18% and 6% were hydroxylated di-acylated and tri-acylated MELs, respectively. As discussed before, the conventional di-MEL structures of *M. aphidis* from castor oil were different to those obtained from rapeseed oil. As a result, a completely different and novel MEL mixture was obtained for this organism. *U. siamensis* and *U. shanxiensis* MELs from castor oil were very similar in their composition, which was already apparent from GC and MALDI-TOF-MS analysis. The main difference between the two was a higher amount of tri-acylated hydroxylated MEL structures with *U. siamensis*, while *U. shanxiensis* had a higher share of di-acylated hydroxylated MELs. Overall, the sum of hydroxylated MEL structures (di- and tri-acylated) in those two species with castor oil accounted for roughly 50% of the mixture and was thus the highest of all investigated organisms.

**Table 3.** Detailed HPLC-ESI-MS Peak Analysis of *Ustilago siamensis* MEL With Castor Oil. Highlighted MEL Structures (*) Were Further Investigated by NMR to Confirm the Structure and Assign the Position of Fatty Acid Residues

Retention time TIC (min)	Molecular mass (Da)	Detected ions (Da)	Peak area ELSD (%)	Derived MEL structure	MEL sub class	Peak area ELSD (%)
21.65	550.6	411; 429; 533; 551; 568	0.3	MEL-D-C2-C14:1-OH	hydroxy-di-MEL	23.4
22.43	590.7	451; 469; 573; 591; 608	0.1	MEL-B/C-C2-C14:2-OH		
22.95	592.7	453; 471; 575; 593; 610	0.2	MEL-B/C-C2-C14:1-OH		
23.72	578.7 (*)	439; 457; 561; 579; 596	7.6	MEL-D-C4-C14:1-OH (*)		
24.84	620.7 (*)	257; 481; 499; 603; 621; 638	3.5	MEL-B/C-C4-C14:1-OH (*)		
25.1	620.7 (*)	257; 481; 499; 603; 621; 638	8.1	MEL-B/C-C4-C14:1-OH (*)		
25.7	606.7	467; 483; 589; 607; 624	1.1	MEL-D-C4-C16:1-OH		
26.56	648.8	509; 525; 631; 649; 666	0.4	MEL-B/C-C4-C16:1-OH		
26.91	648.8	509; 525; 631; 649; 666	2.1	MEL-B/C-C4-C16:1-OH		
28.63	602.7	257; 481; 620	0.9	MEL-B/C-C4-C14:2	di-MEL	56.2
29.92	564.7	257; 443; 582	5.4	MEL-D-C4-C14:0		
30.44	606.7	257; 485; 624	5.3	MEL-B/C-C4-C14:0		
30.87	632.8	257; 511; 650	16.6	MEL-B/C-C4-C16:1		
31.3	634.8	257; 513; 652	11.0	MEL-B/C-C4-C16:0		
31.64	634.8	257; 513; 652	17.0	MEL-B/C-C4-C16:0		
33.88	882.7	257; 481; 761; 900	1.1	MEL-B/C-C4-C14:1-OH-C18:2	hydroxy-tri-MEL	18.3
34.23	842.7	257; 439; 721; 860	4.2	MEL-D-C4-C14:1-OH-C18:1		
34.49	884.7 (*)	257; 481; 763; 902	10.0	MEL-B/C-C4-C14:1-OH-C18:1 (*)		
35.09	912.8	257; 509; 791; 930	3.0	MEL-B/C-C4-C16:1-OH-C18:1		

**Table 4.** Detailed HPLC-ESI-MS Peak Analysis of *Moesziomyces aphidis* MEL With Castor Oil

Retention time TIC (min)	Molecular mass (Da)	Detected ions (Da)	Peak area ELSD (%)	Derived MEL structure	MEL sub class	Peak area ELSD (%)
21.74	550.6	411; 429; 551; 568	0.2	MEL-D-C2-C14:1-OH	hydroxy-di-MEL	18.2
23.03	592.7	453; 471; 575; 593	0.2	MEL-B/C-C2-C14:1-OH		
23.46	592.7	453; 471; 575; 593	0.5	MEL-B/C-C2-C14:1-OH		
23.72	578.7	439; 457; 561; 579; 596	2.7	MEL-D-C4-C14:1-OH		
24.93	620.7	481; 499; 603; 621; 638	1.8	MEL-B/C-C4-C14:1-OH		
25.1	620.7	481; 499; 603; 621; 638	10.0	MEL-B/C-C4-C14:1-OH		
26.99	648.8	257; 429; 649	2.8	MEL-B/C-C4-C16:1-OH		
27.51	536.7	271; 415; 554	0.9	MEL-D-C8:0-C8:0	di-MEL	74.0
28.37	578.7	313; 457; 596	3.3	MEL-B/C-C8:0-C8:0		
28.8	578.7	313; 457; 596	21.8	MEL-B/C-C8:0-C8:0		
29.58	620.7	439; 499; 638	6.6	MEL-A-C8:0-C8:0		
29.92	604.7	339; 483; 622	5.4	MEL-B/C-C8:0-C10:1		
30.44	606.7/646.8	341; 485; 525; 569; 624; 664	7.2	MEL-B/C-C8:0-C10:0/MEL-A-C8:0-C10:1		
30.87	606.7/632.8/648.8	257; 383; 485; 511; 527; 624; 650; 666	12.1	MEL-B/C-C8:0-C10:0/MEL-B/C-C4-C16:1/MEL-A-C8:0-C10:0		
31.3	634.8	257; 513; 652	1.4	MEL-B/C-C4-C16:0		
31.64	634.8	257; 513; 652	15.4	MEL-B/C-C4-C16:0		
32.51	882.7	257; 481; 761; 902	1.3	MEL-B/C-C4-C14:1-OH-C18:2	hydroxy-tri-MEL	6.1
33.8	882.7	257; 481; 761; 900	1.2	MEL-B/C-C4-C14:1-OH-C18:2		
34.49	884.7	257; 481; 763; 902	3.0	MEL-B/C-C4-C14:1-OH-C18:1		
35.09	912.8	509; 791; 930	0.6	MEL-B/C-C4-C16:1-OH-C18:1		

### NMR Analysis of Novel Di- and Tri-Acylated Hydroxy-MELs

**Fig. 5.**
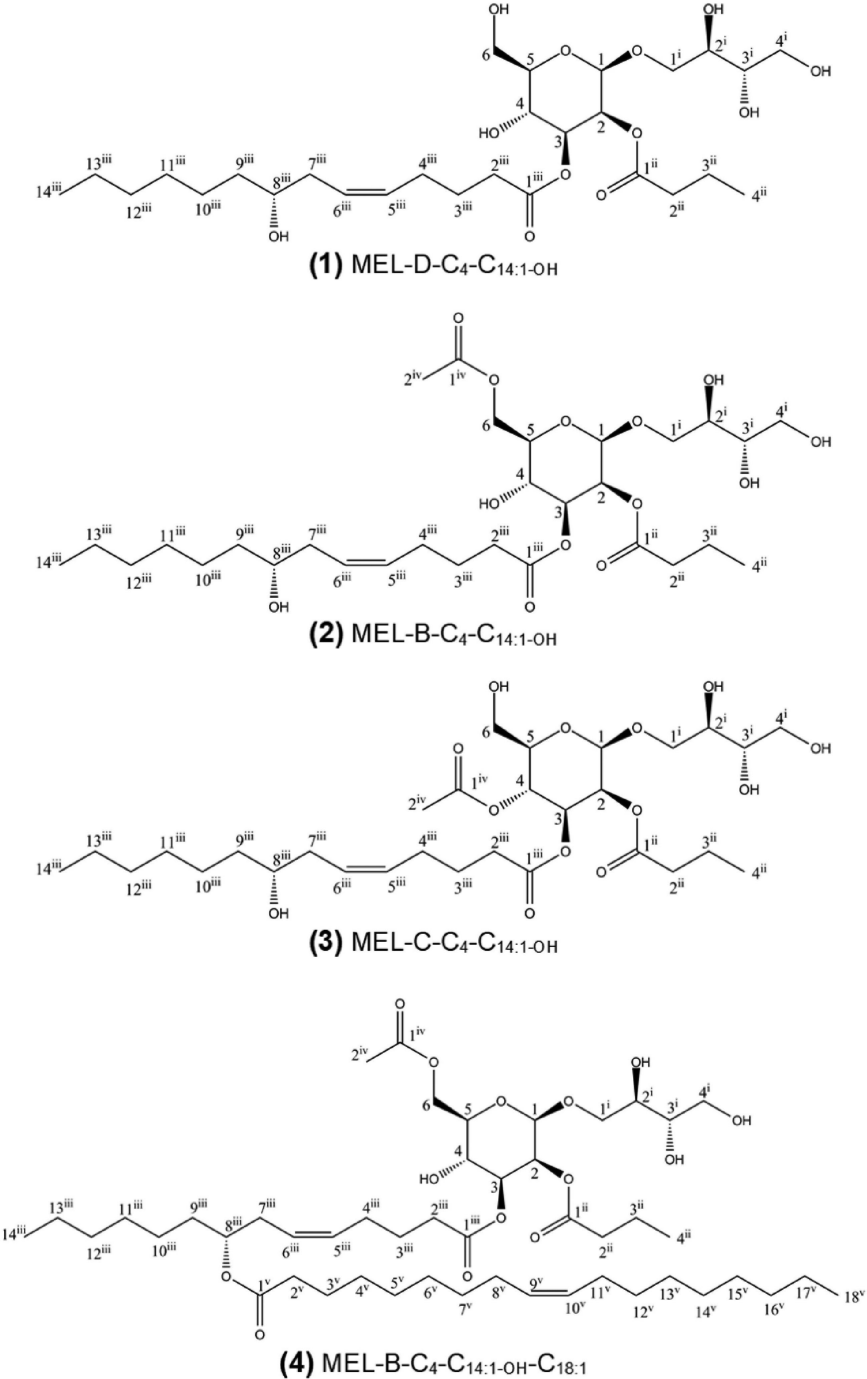
Selected structures of hydroxy-MELs isolated from *Ustilago siamensi*s with castor oil and analyzed by NMR.

Ultimately, NMR analysis of single hydroxy-MELs from *U. siamensis* with castor oil was conducted to verify the novel di- and tri-acylated structures that were deduced based on GC, MALDI-TOF-MS, and HPLC-ESI-MS. Four new hydroxy-MELs, obtained by preparative reversed-phase HPLC and subsequent fractionation, were selected for detailed 1D- and 2D-NMR analysis. In contrast to LC-MS, NMR allows the unambiguous determination of the substitution pattern and positions of each acyl ester residue. The investigated structures **1** to **4** are shown in Fig. 5. Detailed NMR assignments of the four compounds are given in the Supplementary Tables S10–S13.

According to a molecular weight of 578 Da and corresponding LC-MS fragmentation pattern, compound **1** was identified as MEL-D esterified with a butanoic acid (C_4_) and a C_14:1-OH_ fatty acid (8-hydroxy-5-cis-tetradecenoic acid). Methyl group signals together with HH-COSY confirmed that both alkyl residues are n-alkyl residues without any branches. Low-field shift in ^1^H-NMR of protons H-2 and H-3 of the mannose moiety showed that O-2 and O-3 of the mannose are esterified. Correlation in HMBC from H-2 of mannose to C-1^ii^ of the butanoic acid residue and from H-3 to C-1^iii^ of the C_14:1-OH_ acid allowed the elucidation of the fatty acid substitution pattern in compound **1**. This compound was subsequently identified as MEL-D-C_4_-C_14:1-OH_, with the short C_4_ fatty acid at C2′ and the longer C_14:1-OH_ fatty acid at C3′ of the mannose moiety. The position of the additional acetate in compound **2** (molecular mass of 620 Da) was assigned by the chemical shifts of H-6_a_ (4.28 ppm) and H-6_b_ (4.46 ppm) of the mannose together with HMBC-correlation from H-6 to C-1^iv^ of the acetate. The compound was thus identified as MEL-B-C_4_-C_14:1-OH_. The chemical shifts of this compound (see Supplementary Table S11) were mostly similar to the novel MEL-B reported previously by Yamamoto et al. (2013), which was MEL-B-C_8_-C_14:1-OH_. In compound **3**, the acetate group is located at O-4 of the mannose from the shift of H-4 at 5.23 ppm and the compound was subsequently identified as MEL-C-C_4_-C_14:1-OH_. Compound **4** (molecular weight of 884 Da) has an additional C_18:1_ ester compared to compounds **2** and **3**. Positions O-2, O-3, and O-6 of the mannose are esterified, whereas O-4 is not. While the three compounds **1–3** showed ^13^C and ^1^H NMR shifts of 71.9 and 3.60 ppm at the 8^iii^ position of the C_14:1-OH_ residue, which are common for a nonesterified proton of an OH-group in a linear alkyl chain (see also Yamamoto et al., 2013), compound **4** presents different chemical shifts of 73.7 and 4.90 ppm, respectively. This downfield change in chemical shift correlates with the O-acylation of the hydroxy group, proofing that the additional fatty acid ester must be attached to the 8-hydroxy group of the C_14:1-OH_ acid. Compound **4** was thus identified as tri-acylated MEL-B-C_4_-C_14:1-OH_-C_18:1_.

## Discussion

In the current study, we investigated for the first time MEL production from castor oil using seven different *Ustilaginaceae* species in a systematic approach. The aim of the work was to test castor oil as a possible substrate and to evaluate if novel MEL structures could be produced from its unconventional fatty acids. The resulting MEL yields from castor oil were in the low g/l range and were thus lower than for other plant oils like soybean or rapeseed oil. Nevertheless, production of MEL from castor oil might still be interesting, since we could detect some novel MEL structures, which had not been observed before with other plant oils. In general, two novel MEL types were observed. These were (1) di-acylated MELs containing a hydroxy fatty acid and (2) so-called tri-acylated MELs, where a third fatty acid is esterified to the hydroxyl group of the fatty acid side chain.

Di-acylated MELs with a hydroxy fatty acid had been shown before by Yamamoto et al. (2013). While they only reported production of those novel hydroxylated di-MELs from castor oil with the species *P. tsukubaensis* NBRC1940, our present work shows that integration of hydroxyl fatty acids into MEL could be a general feature of multiple *Ustilaginaceae* species. Especially *M. aphidis* DSM70725, *U. siamensis* CBS 9960, and *U. shanxiensis* CBS 10075 were shown to produce substantial shares of hydroxylated MEL structures from castor oil under the given cultivation conditions.

It is generally accepted that the fatty acid side chains in MEL are derived from the so-called peroxisomal chain-shortening pathway, which uses a partial β-oxidation to produce medium-chain fatty acids. This is done by repeated removal of C2-units from the carboxy-terminal end of the fatty acids (Kitamoto et al., 1998). In studies with FAME as substrates, Kitamoto et al. (1995) and Rau et al. (2005) could show for *M. antarcticus* and *M. aphidis*, respectively, that even-chain FAMEs led to even-chain fatty acids in MEL, while odd-chain FAMEs led to odd-chain fatty acids in MEL. Additionally, cerulenin, a blocker of *de-novo* fatty acid synthesis, did not have an effect on MEL production in *M. antarcticus*, further supporting the chain-shortening hypothesis (Kitamoto et al., 1995). Now, when using castor oil as substrate, of which ricinoleic acid (C_18:1-OH_) is the major fatty acid, it can be expected that even-chain hydroxy fatty acids are generated during chain-shortening, that is, C_16:1-OH_, C_14:1-OH_, and so on. Our gas chromatographic analysis indeed showed the presence of hydroxylated fatty acids in the MELs from castor oil of all seven investigated species. Those were mainly C_14:1-OH_ and to a lesser extent C_16:1-OH_. Hydroxy fatty acids with a lower number of carbon atoms, for example, C_12:1-OH_ or C_10:1-OH_, were not found, even though some of the species have a preference for shorter fatty acids in their MELs.

For *U. siamensis* and *U. shanxiensis*, the production of novel hydroxylated MEL-B/C-C_4_-C_16:1-OH_ or MEL-B/C-C_4_-C_14:1-OH_ from castor oil is consistent with the usual chain length of fatty acids from other oils. The MELs of *U. siamensis* and *U. shanxiensis* usually contain a short-chain (C_2_ or C_4_) and a long-chain (C_14_-C_18_) fatty acid when fed with soybean or rapeseed oil. Depending on the degree of saturation in the substrate oil, a high share of unsaturated fatty acids is found (Beck et al., 2019). Similarly, Yamamoto et al. (2013) observed production of MEL-B-C_8_-C_14:1-OH_ with *P. tsukubaensis* and castor oil, which is also in agreement with the fatty acid chain length of this species from other oils (Beck et al., 2019; Fukuoka et al., 2008). Contrary to that we found that *M. aphidis*, which usually produces MELs with a chain length of 8 or 10 carbon atoms for both fatty acids (Beck et al., 2019; Onghena et al., 2011; Rau et al., 2005), produced high shares of hydroxylated MEL-B/C-C_4_-C_14:1-OH_ from castor oil. This is thus very different to the usual fatty acid chain length from other plant oils with *M. aphidis*. Moreover, the conventional nonhydroxylated di-MELs in the product mixture of *M. aphidis* from castor oil were also different to the typical di-MEL pattern from other oils. Here we could detect MEL-B/C-C_8_-C_8_ and MEL-B/C-C_4_-C_16_ when using castor oil as substrate, while from rapeseed or soybean oil mostly MEL-A-C_8_-C_10_ and MEL-A-C_10_-C_10_ were produced by *M. aphidis*.

All these observations actually raise the question—Which principle is governing the chain length of fatty acids in MELs from castor oil? It could be either the substrate specificity of the two acyltransferases (Mac1 and Mac2) or the termination of chain-shortening pathway at a specific carbon chain length. Since the chain-shortening pathway and the acylation reaction of mannosylerythritol by Mac1 and Mac2 are both located in peroxisomes (Freitag et al., 2014), there is most likely an interdependence of both mechanisms. Using cross-species complementation of the respective genes, Deinzer et al. (2019) showed that the catalytic activity of the enzymes Mat1, Mac1, and Mac2 determines the specific MEL pattern in *U. maydis* and *U. hordei* (Deinzer et al., 2019). It can thus be assumed that this holds true also for other *Ustilaginaceae* species. Lately, the same group also succeeded to construct a *U. maydis* mutant strain expressing different Mac1 and Mac 2 genes from other species (Becker et al., 2021). These mutant strains were producing completely novel and tailor-made combinations of fatty acid side chains, where chain length was only governed by the substrate specificities of the two inserted acyltransferases. Besides chain length, also the position of the two fatty acid residues was shown to be determined by enzyme specificity, where Mac1 catalyzed addition of the shorter fatty acid at C2′ and Mac2 addition of the longer fatty acid at C3′. (Becker et al., 2021). However, when using hydroxylated fatty acids as substrate as we did here, it seems that chain shortening already terminates at C_14:1-OH_. This could be due to the special conformation of hydroxy fatty acids, which prevents them from further degradation. These fatty acids are then integrated into the MELs regardless of the usual enzyme specificities. Otherwise, the occurrence of MEL-C_4_-C_14:1-OH_ in *M. aphidis*, for example, could not be explained. As a result, we concluded that the chain-shortening also plays a significant role for the chain length of fatty acid residues in MEL, besides enzyme specificity of Mac1 and Mac2.

Another possible pathway for the supply of fatty acids might also be *de-novo* fatty acid synthesis from acetyl-CoA, which has been shown to be active when water-soluble substrates like sugars or glycerol are used as sole substrate instead of plant oils (Morita et al., 2007; Wada et al., 2020). In contrast to the chain-shortening mechanism, *de-novo* synthesis produces mostly saturated fatty acids. It has been shown that the fatty acid profile of *M. antarcticus* T-34 MELs from glucose contained more saturated fatty acids than those from soybean oil (Morita et al., 2007). This might also explain the occurrence of saturated fatty acids in our experiments with castor oil, although they are not present in the substrate originally. *De-novo* synthesis of fatty acids might be activated when the organisms are not able to generate enough energy from chain shortening of the castor oil fatty acids, which is terminating already at C_14:1-OH_. Most likely, a combination of both pathways (β-oxidation of hydroxy fatty acids and de-novo synthesis of saturated acids from acetyl-CoA) actually leads to the respective fatty acid pattern in MELs from castor oil, but further research on the detailed metabolic pathways needs to be done.

Lastly, the detection of tri-acylated MEL structures with a third fatty acid esterified to the free hydroxyl group was also quite remarkable. Similar to ‘conventional’ tri-acylated MELs, where the third fatty acid is bound to the primary hydroxyl group of erythritol (Fukuoka et al., 2007a; Goossens et al., 2016; Morita et al., 2008), it can be assumed that those structures result from enzyme-catalyzed reaction of free fatty acids with the novel hydroxyl MELs in the culture broth. The position of the third fatty acid was, however, very unusual. As we were able to show by detailed fragmentation analysis in LC-MS and NMR spectroscopy, those novel tri-MELs have the third fatty acid esterified to the hydroxyl group of the C_14:1-OH_ or C_16:1-OH_ hydroxy fatty acid. Such a structure has never been observed before. Thus, the additional hydroxyl group in the novel MELs from castor oil can be seen as a good starting point for further modification, leading to completely new MEL structures. Although this is fascinating, the reproducibility of these tri-MEL structures might be challenging. Since they are a result of lipase-catalyzed reaction with free fatty acids in the culture broth, their formation is strongly dependent on culture conditions and thus could be different between batches. This challenge, however, might be tackled by improvements in the cultivation procedure. Alternatively, lipase-catalyzed esterification of all hydroxy-MELs with an additional fatty acid could be done intentionally after purification, in order to obtain only tri-acylated hydroxy-MELs. A similar approach but for conventional MEL structures was, for example, published by Fukuoka et al. (2007a) or Recke et al. (2013).

## Conclusion

With the current work, we have been able to show that production of MELs from castor oil is generally possible with a variety of microorganisms from the *Ustilaginaceae* family. Although the yields were only in the low g/l range, we could demonstrate that several new and interesting MEL structures were produced from this substrate. These novel structures, which were di-acylated MELs containing a mono-unsaturated hydroxylated fatty acid (C_14:1-OH_ or C_16:1-OH_) or tri-acylated MELs with an additional long-chain fatty acid (C_16_ or C_18_), should have different properties and can therefore be exploited for new applications. The most promising producers of these novel structures in our screening were *U. siamensis* CBS 9960, *U. shanxiensis* CBS 10075, and *M. aphidis* DSM70725. By further optimizing the cultivation procedure for this substrate, higher MEL titers could be achieved that will provide exciting new MEL mixtures with new and interesting properties.

## Supplementary Material

kuab042_Supplemental_File
