# Supplementary material for: Novel mannosylerythritol lipid biosurfactant structures from castor oil revealed by advanced structure analysis
Source: J Ind Microbiol Biotechnol. 2021 Jul 29;48(7-8):kuab042. doi: 10.1093/jimb/kuab042 (PMC8788835; doi:10.1093/jimb/kuab042)
Supplement: kuab042_Supplemental_File [file kuab042_supplemental_file.docx]

**Journal of Industrial Microbiology and Biotechnology**

**Novel mannosylerythritol lipid biosurfactant structures from castor oil revealed by advanced structure analysis**

Alexander Beck^1,3^, Fabian Haitz ^3,+^, Isabel Thier^2^, Karsten Siems^2^, Sven Jakupovic^2^, Steffen Rupp^3^, Susanne Zibek^1,3,*^

^1^ Institute of Interfacial Process Engineering and Plasma Technology IGVP, University of Stuttgart, Stuttgart, Germany

^2^ AnalytiCon Discovery GmbH, Potsdam, Germany

^3^ Fraunhofer Institute for Interfacial Engineering and Biotechnology IGB, Stuttgart, Germany

^+^ Currently: Department of Mechanical and Process Engineering, Offenburg University, Offenburg, Germany

**Contact details**

Alexander Beck, Fraunhofer Institute for Interfacial Engineering and Biotechnology IGB,
Nobelstraße 12, 70569 Stuttgart, Germany, +49 711 970-4080, [alexander.beck@igb.fraunhofer.de](mailto:alexander.beck@igb.fraunhofer.de), ORCID: 0000-0002-4860-0334

* Corresponding author: Susanne Zibek, Fraunhofer Institute for Interfacial Engineering and Biotechnology IGB,
Nobelstraße 12, 70569 Stuttgart, Germany, +49 711 970-4167, [susanne.zibek@igb.fraunhofer.de](mailto:susanne.zibek@igb.fraunhofer.de), ORCID: 0000-0001-5344-6549

**Fig. S1** HPTLC analysis of culture broth extracts from cultivations with castor oil and rapeseed oil for the seven *Ustilaginaceae* species. Pure castor oil and castor oil hydrolysis products as well as ricinoleic acid are shown as reference substances.


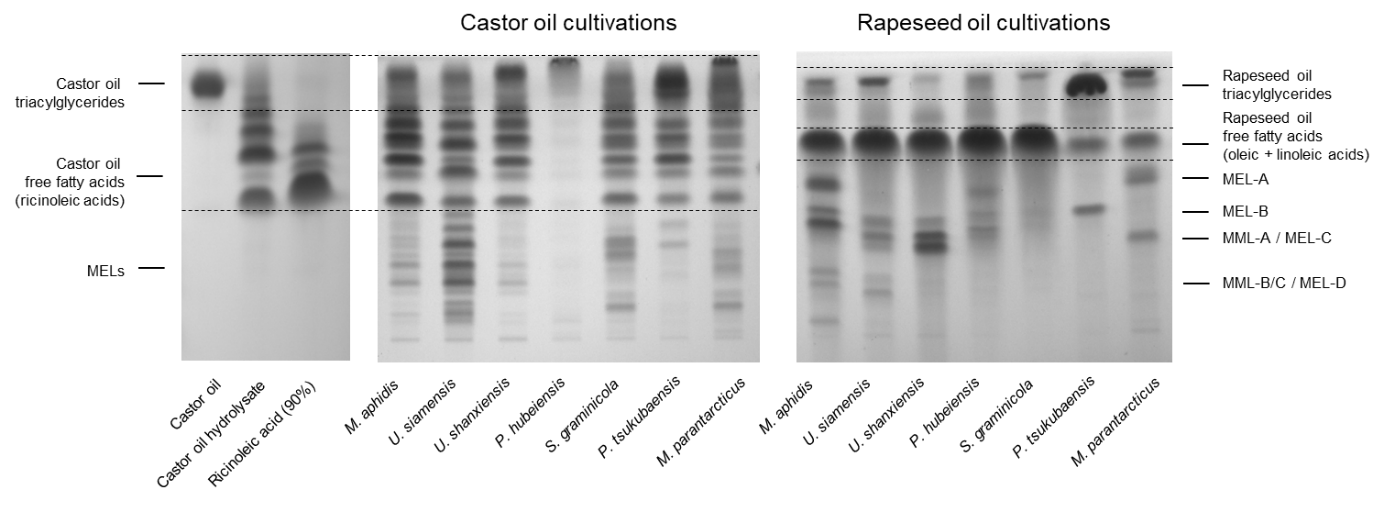


**Fig. S2** Combined HPTLC and MALDI-TOF-MS analysis of purified MELs from *U. siamensis* with rapeseed oil (a-c), *U. siamensis* with castor oil (d-f) and *M. aphidis* with castor oil (g-i). After HPTLC separation of MEL mixtures on silica gel (a,d,g), 2-dimensional mass spectra were generated by MALDI-TOF mass spectrometry (b,e,h). For each spot in the mass spectrum, the resulting ions were then compared to previously calculated theoretical masses and gas chromatographic results to deduce the molecular structures of MEL congeners (c,f,i). The MELs from castor oil showed novel ions for example at m/z 643.5 and 671 [M+Na]+ that could be linked to MEL-B/C structures containing a hydroxy fatty acid.


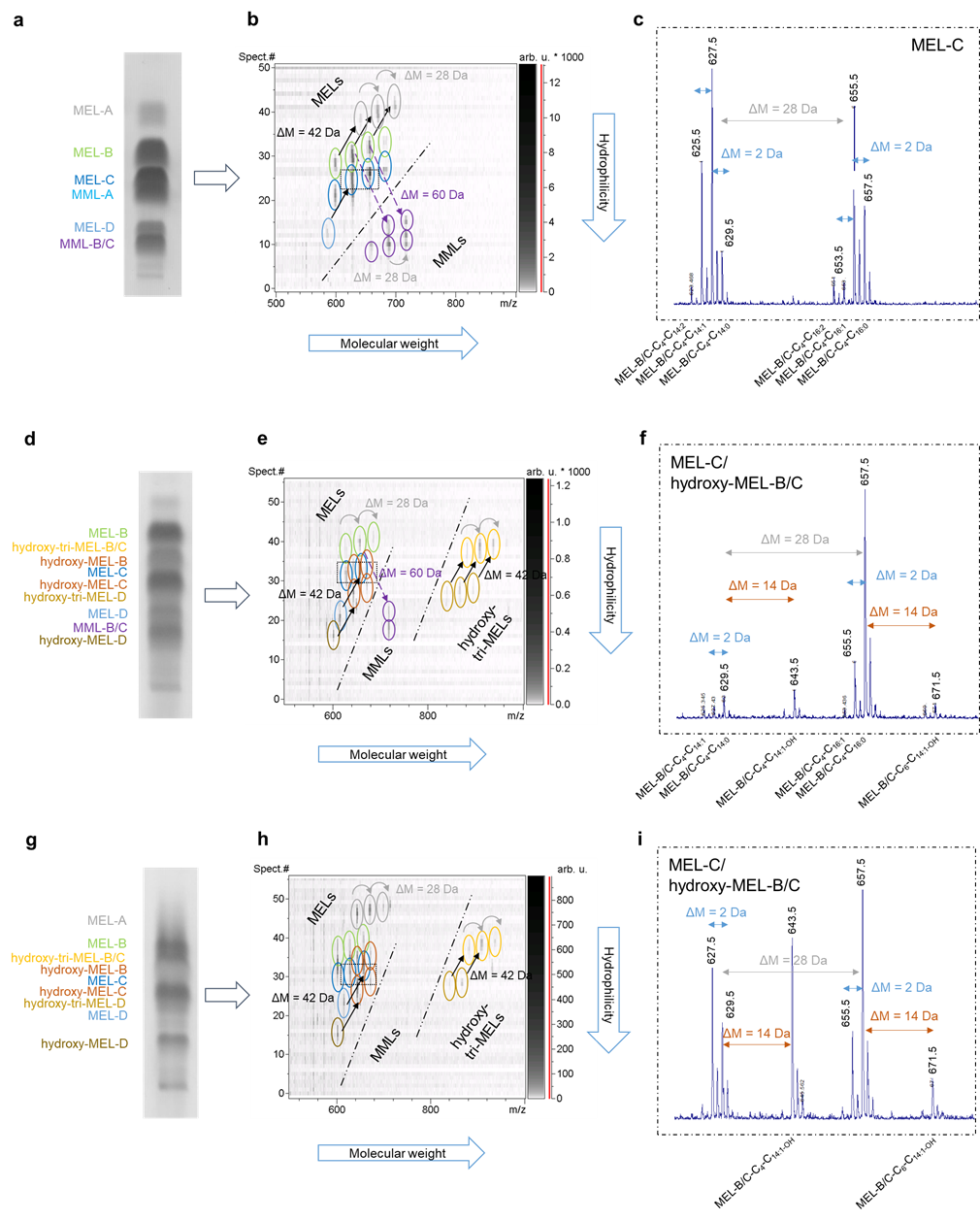


**Fig. S3** HPLC-ELSD chromatograms of *M. aphidis* MEL from rapeseed oil (upper) and castor oil (lower). Resulting peaks are categorized into conventional di-acylated MELs (di-MELs), novel di-acylated MELs with hydroxylated fatty acids (hydroxy-di-MELs) and tri-acylated MELs with hydroxylated fatty acids (hydroxy-tri-MELs).

**
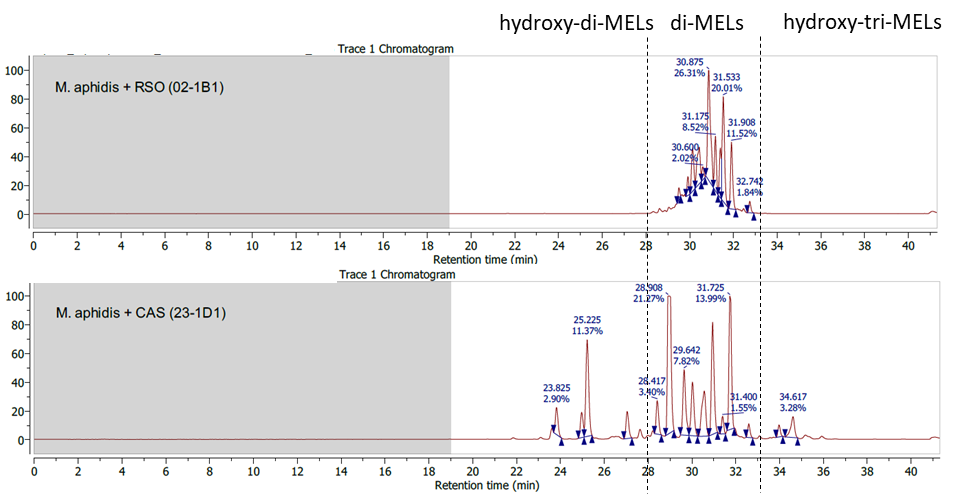
**

**Fig. S4** HPLC-ELSD chromatograms of *U. siamensis* MEL with rapeseed oil (upper) and castor oil (lower). Resulting peaks are categorized into conventional di-acylated MELs (di-MELs), novel di-acylated MELs with hydroxylated fatty acids (hydroxy-di-MELs) and tri-acylated MELs with hydroxylated fatty acids (hydroxy-tri-MELs).


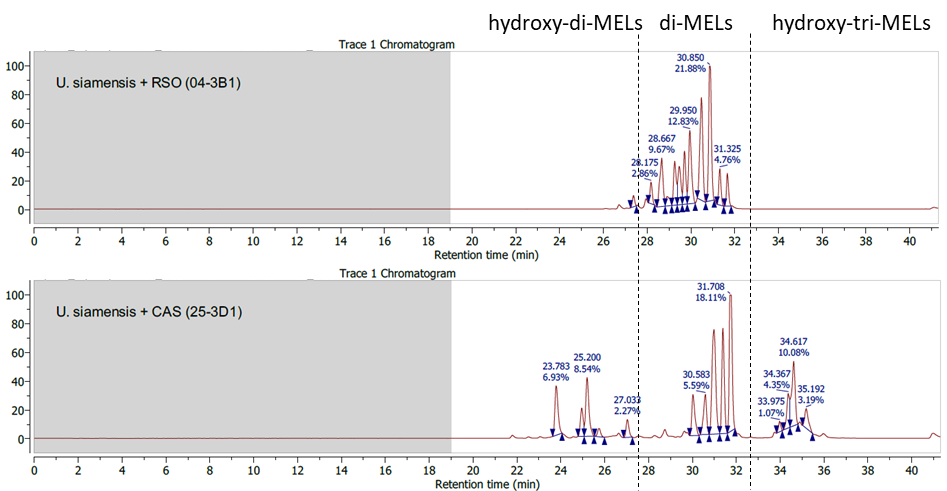


**Figure S5** HPLC-ELSD chromatograms of *U. shanxiensis* MEL from rapeseed oil (upper) and castor oil (lower). Resulting peaks are categorized into conventional di-acylated MELs (di-MELs), novel di-acylated MELs with hydroxylated fatty acids (hydroxy-di-MELs) and tri-acylated MELs with hydroxylated fatty acids (hydroxy-tri-MELs).


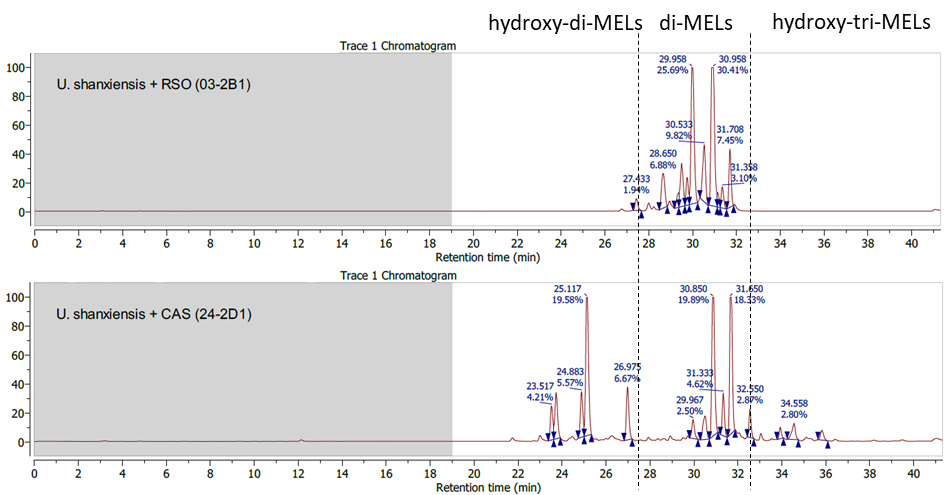


**Figure S6** HPLC-ELSD chromatograms of *M. parantarcticus* MEL from rapeseed oil (upper) and castor oil (lower). Resulting peaks are categorized into conventional di-acylated MELs (di-MELs), novel di-acylated MELs with hydroxylated fatty acids (hydroxy-di-MELs) and tri-acylated MELs with hydroxylated fatty acids (hydroxy-tri-MELs).


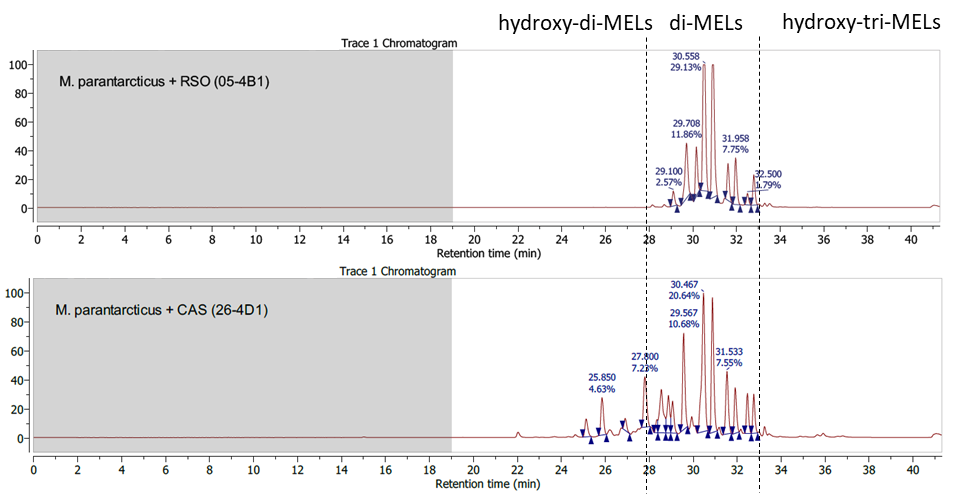


**Figure S7** HPLC-ELSD chromatograms of *S. graminicola* MEL from rapeseed oil (upper) and castor oil (lower). Resulting peaks are categorized into conventional di-acylated MELs (di-MELs), novel di-acylated MELs with hydroxylated fatty acids (hydroxy-di-MELs) and tri-acylated MELs with hydroxylated fatty acids (hydroxy-tri-MELs).


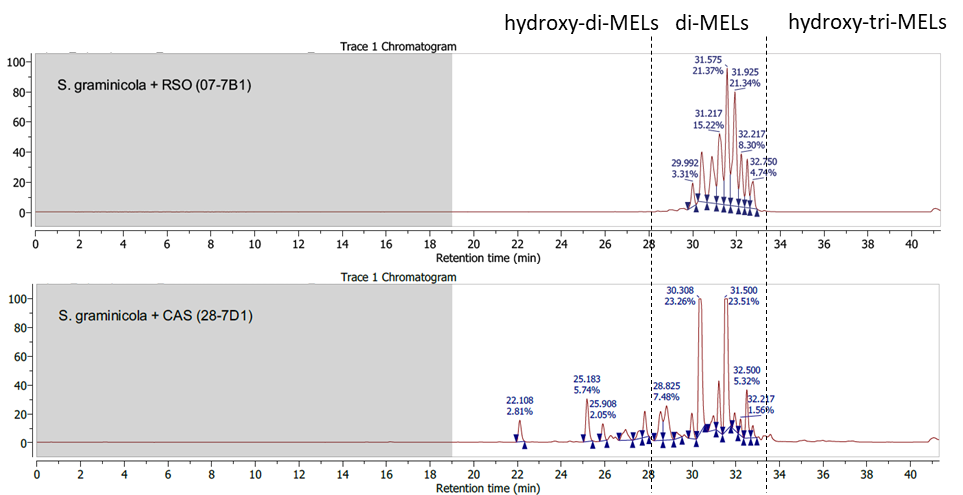


**Figure S8** HPLC-ELSD chromatograms of *P. tsukubaensis* MEL from rapeseed oil (upper) and castor oil (lower). Resulting peaks are categorized into conventional di-acylated MELs (di-MELs), novel di-acylated MELs with hydroxylated fatty acids (hydroxy-di-MELs) and tri-acylated MELs with hydroxylated fatty acids (hydroxy-tri-MELs).


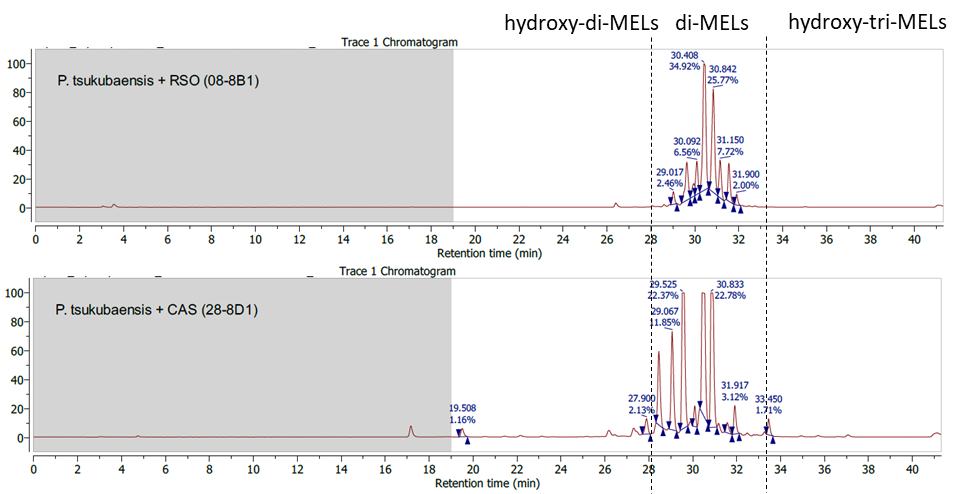


**Table S9** Detailed HPLC-ESI-MS peak analysis of *U. shanxiensis* MEL with castor oil.

| **Retention time  TIC (min)** | **molecular mass  (Da)** | **detected ions (Da)** | **peak area  ELSD (%)** | **derived MEL structure** | **MEL  sub class** | **peak area  ELSD (%)** |
| --- | --- | --- | --- | --- | --- | --- |
| 21.65 | 550.6 | 411; 429; 533; 551; 568 | 0.3 | MEL-D-C2-C14:1-OH | hydroxy-di-MEL | 41.5 |
| 22.86 | 592.7 | 453; 471; 575; 593; 610 | 0.8 | MEL-B/C-C2-C14:1-OH |  |  |
| 23.46 | 592.7 | 453; 471; 575; 593; 610 | 4.0 | MEL-B/C-C2-C14:1-OH |  |  |
| 23.63 | 578.7 | 421; 439; 457; 561; 579; 596 | 5.8 | MEL-D-C4-C14:1-OH |  |  |
| 24.24 | 606.7 | 467; 485; 589; 607; 624 | 0.7 | MEL-D-C4-C16:1-OH |  |  |
| 24.75 | 620.7 | 257; 499; 603; 621; 648 | 5.3 | MEL-B/C-C4-C14:1-OH |  |  |
| 25.01 | 620.7 | 257; 499; 603; 621; 648 | 18.5 | MEL-B/C-C4-C14:1-OH |  |  |
| 26.91 | 648.8 | 285; 429; 568; 649 | 6.3 | MEL-B/C-C4-C16:1-OH |  |  |
| 29.84 | 604.7 | 229; 423; 483; 622 | 2.7 | MEL-B/C-C4-C14:1 | di-MEL | 46.9 |
| 30.44 | 606.7 / 632.8 | 257; 485; 511; 624; 650 | 3.9 | MEL-B/C-C4-C14:0 / MEL-B/C-C4-C16:1 |  |  |
| 30.78 | 606.7 / 632.8 | 257; 485; 511; 624; 650 | 18.8 | MEL-B/C-C4-C14:0 / MEL-B/C-C4-C16:1 |  |  |
| 31.21 | 634.8 | 257; 513; 652 | 4.4 | MEL-B/C-C4-C16:0 |  |  |
| 31.56 | 634.8 | 257; 513; 652 | 17.3 | MEL-B/C-C4-C16:0 |  |  |
| 32.42 | 882.7 | 257; 481; 761; 779; 884; 901; 918 | 2.7 | MEL-B/C-C4-C14:1-OH-C18:2 | hydroxy-tri-MEL | 7.3 |
| 33.8 | 882.7 | 257; 481; 761; 883; 900 | 1.1 | MEL-B/C-C4-C14:1-OH-C18:2 |  |  |
| 34.49 | 884.7 | 257; 481; 763; 885; 902 | 2.6 | MEL-B/C-C4-C14:1-OH-C18:1 |  |  |
| 35.09 | 886.7 / 912.8 | 257; 509; 765; 791; 904; 930 | 0.8 | MEL-B/C-C4-C14:1-OH-C18:0 / MEL-B/C-C4-C16:1-OH-C18:1 |  |  |

**Table S10** NMR analysis of *U. siamensis* MEL-D-C_4_-C_14:1-OH_ (hydroxy-di-MEL) produced from castor oil.

|  | | *U. siamensis* + castor oil | | | | |
| --- | --- | --- | --- | --- | --- | --- |
| **MEL type** | | MEL-D-C_4_-C_14:1-OH_ | | | | |
| **Structure** | | 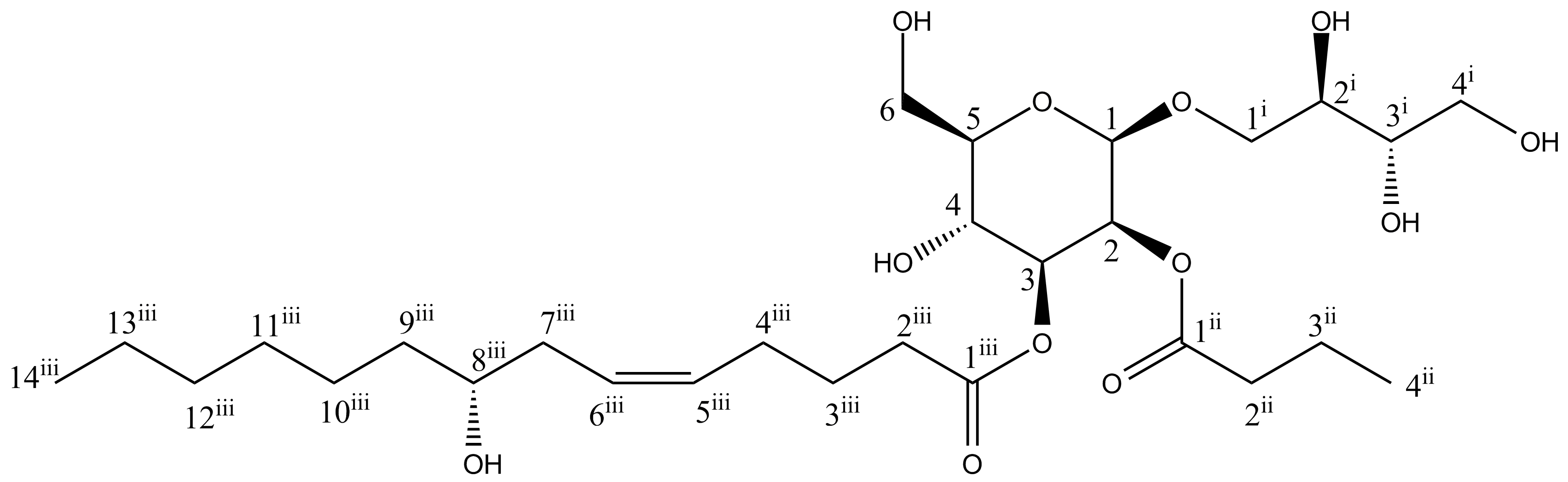 | | | | |
| Position | δ_C_ [ppm] | *multiplicity* | δ_H_ [ppm] | *multiplicity* | INT | J [Hz] |
| 1 | 98.9 | *d* | 4.85 | *br s* |  |  |
| 2 | 69.3 | *d* | 5.48 | *m* |  |  |
| 3 | 73.9 | *d* | 4.93 | *m* |  |  |
| 4 | 64.7 | *d* | 3.77 | *m* |  |  |
| 5 | 76.9 | *d* | 3.41 | *m* |  |  |
| 6 | 61.2 | *t* | 3.77  3.93 | *m**  *dd* |  | -  2.3, 11.7 |
| 1^i^ | 71.2 | *t* | 3.70  4.08 | *m*  *m* |  |  |
| 2^i^ | 71.9 | *d* | 3.57 | *m* |  |  |
| 3^i^ | 71.2 | *d* | 3.57 | *m* |  |  |
| 4^i^ | 63.2 | *t* | 3.60  3.73 | *m**  *m** |  |  |
| 1^ii^ | 173.3 | *s* | - |  |  |  |
| 2^ii^ | 35.6 | *t* | 2.39 | *t* | 2H | 7.3 |
| 3^ii^ | 18.2 | *t* | 1.70 |  | 2H |  |
| 4^ii^ | 12.7 | *q* | 1.01 | *t* | 3H | 7.5 |
| 1^iii^ | 173.0 | *s* | - |  |  |  |
| 2^iii^ | 33.0 | *t* | 2.32 | *m* | 2H |  |
| 3^iii^ | 24.3 | *t* | 1.68 | *m* | 2H |  |
| 4^iii^ | 26.2 | *t* | 2.12 | *m* | 2H |  |
| 5^iii^ | 130.0 | *d* | 5.47 | *m** |  |  |
| 6^iii^ | 126.7 | *d* | 5.50 | *m** |  |  |
| 7^iii^ | 34.8 | *t* | 2.22 | *m* | 2H |  |
| **8^iii^** | **71.0** | ***d*** | **3.57** | ***m**** |  |  |
| 9^iii^ | 36.4 | *t* | 1.41  1.48 | *m**  *m** |  |  |
| 10^iii^ | 25.4 | *t* | 1.34  1.48 | *m**  *m** |  |  |
| 11^iii^ | 29.2 | *t* | 1.35 | *m* | 2H |  |
| 12^iii^ | 31.6 | *t* | 1.33 | *m* | 2H |  |
| 13^iii^ | 22.3 | *t* | 1.34 | *m* | 2H |  |
| 14^iii^ | 12.9 | *q* | 0.93 | *t* | 3H | 7.0 |

- * Overlapping signal
- Bruker TOPSPIN 4.0.9; 400 MHz (Solvent: CD_3_OD; δ_C_ = 48.0 ppm; δ_H_ = 3.33 ppm)

**Table S11** NMR analysis of *U. siamensis* MEL-B-C_4_-C_14:1-OH_ (hydroxy-di-MEL) produced from castor oil.

|  | | *U. siamensis* + castor oil | | | | |
| --- | --- | --- | --- | --- | --- | --- |
| **MEL type** | | MEL-B-C_4_-C_14:1-OH_ | | | | |
| **Structure** | | 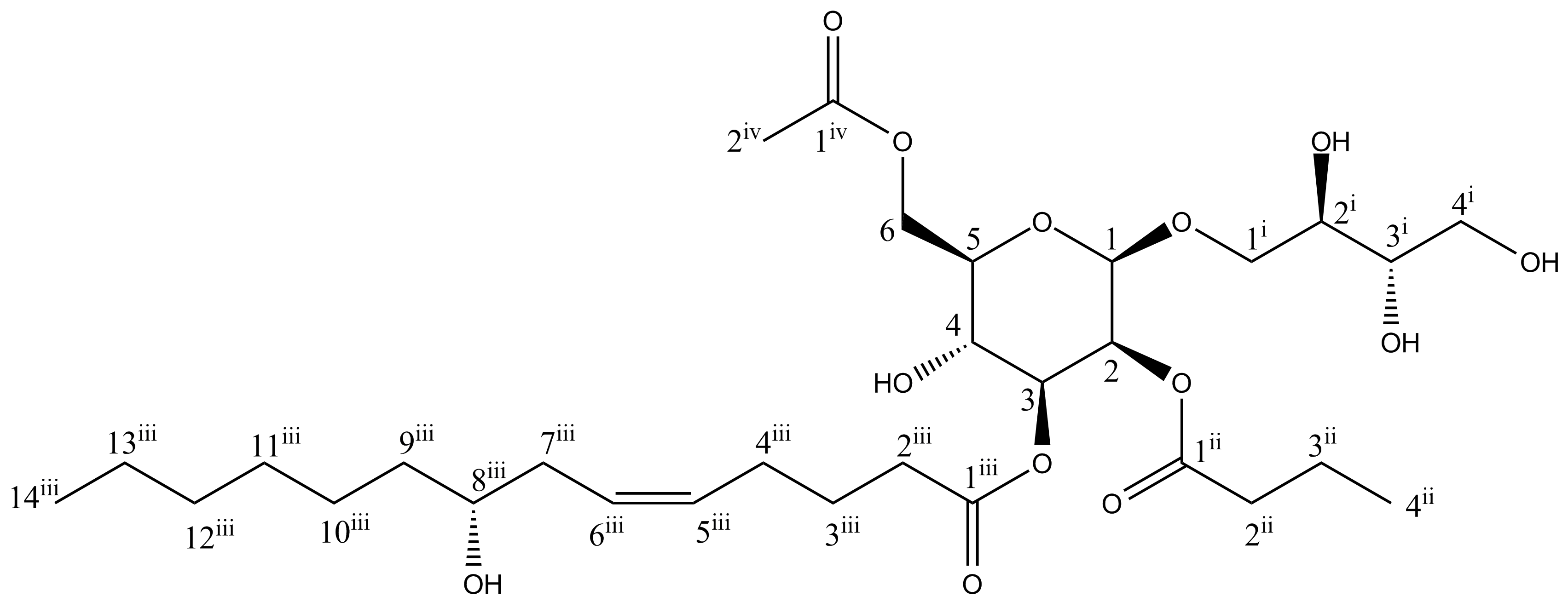 | | | | |
| Position | δ_C_ [ppm] | *multiplicity* | δ_H_ [ppm] | *multiplicity* | INT | J [Hz] |
| 1 | 99.1 | *d* | 4.87 | *br s* |  |  |
| 2 | 69.1 | *d* | 5.49 | *m** |  |  |
| 3 | 73.4 | *d* | 4.95 | *dd* |  | 3.0, 9.5 |
| 4 | 72.1 | *d* | 3.77 | *m* |  |  |
| 5 | 74.1 | *d* | 3.62 | *m* |  |  |
| 6 | 63.2 | *t* | 4.28  4.46 | *m**  *dd* |  | 6.0, 11.9  2.2, 11.9 |
| 1^i^ | 71.9 | *t* | 3.68  4.05 | *m*  *m* |  |  |
| 2^i^ | 71.0 | *d* | 3.57 | *m* |  |  |
| 3^i^ | 72.2 | *d* | 3.58 | *m* |  |  |
| 4^i^ | 63.0 | *t* | 3.59  3.74 | *m**  *m** |  |  |
| 1^ii^ | 173.3 | *s* | - |  |  |  |
| 2^ii^ | 35.6 | *t* | 2.40 | *t* | 2H | 7.2 |
| 3^ii^ | 18.1 | *t* | 1.70 |  | 2H |  |
| 4^ii^ | 12.5 | *q* | 1.02 | *t* | 3H | 7.4 |
| 1^iii^ | 173.0 | *s* | - |  |  |  |
| 2^iii^ | 32.9 | *t* | 2.33 | *m* | 2H |  |
| 3^iii^ | 24.4 | *t* | 1.67 | *m* | 2H |  |
| 4^iii^ | 26.3 | *t* | 2.11 | *m* | 2H |  |
| 5^iii^ | 129.9 | *d* | 5.47 | *m** |  |  |
| 6^iii^ | 126.6 | *d* | 5.49 | *m** |  |  |
| 7^iii^ | 34.9 | *t* | 2.21 | *m* | 2H |  |
| **8^iii^** | **71.9** | ***d*** | **3.59** | ***m**** |  |  |
| 9^iii^ | 36.4 | *t* | 1.40  1.47 | *m**  *m** |  |  |
| 10^iii^ | 25.4 | *t* | 1.34  1.47 | *m**  *m** |  |  |
| 11^iii^ | 29.1 | *t* | 1.33 | *m* | 2H |  |
| 12^iii^ | 31.6 | *t* | 1.32 | *m* | 2H |  |
| 13^iii^ | 22.3 | *t* | 1.33 | *m* | 2H |  |
| 14^iii^ | 13.0 | *q* | 0.92 | *t* | 3H | 7.0 |
| 1^iv^ | 171.3 | *s* | - |  |  |  |
| 2^iv^ | 19.3 | *q* | 2.10 | *s* | 3H |  |

- * Overlapping signal
- Bruker TOPSPIN 4.0.9; 400 MHz (Solvent: CD_3_OD; δ_C_ = 48.0 ppm; δ_H_ = 3.33 ppm)

**Table S12** NMR analysis of *U. siamensis* MEL-C-C_4_-C_14:1-OH_ (hydroxy-di-MEL) produced from castor oil.

|  | | *U. siamensis* + castor oil | | | | |
| --- | --- | --- | --- | --- | --- | --- |
| **MEL type** | | MEL-C-C_4_-C_14:1-OH_ | | | | |
| **Structure** | | 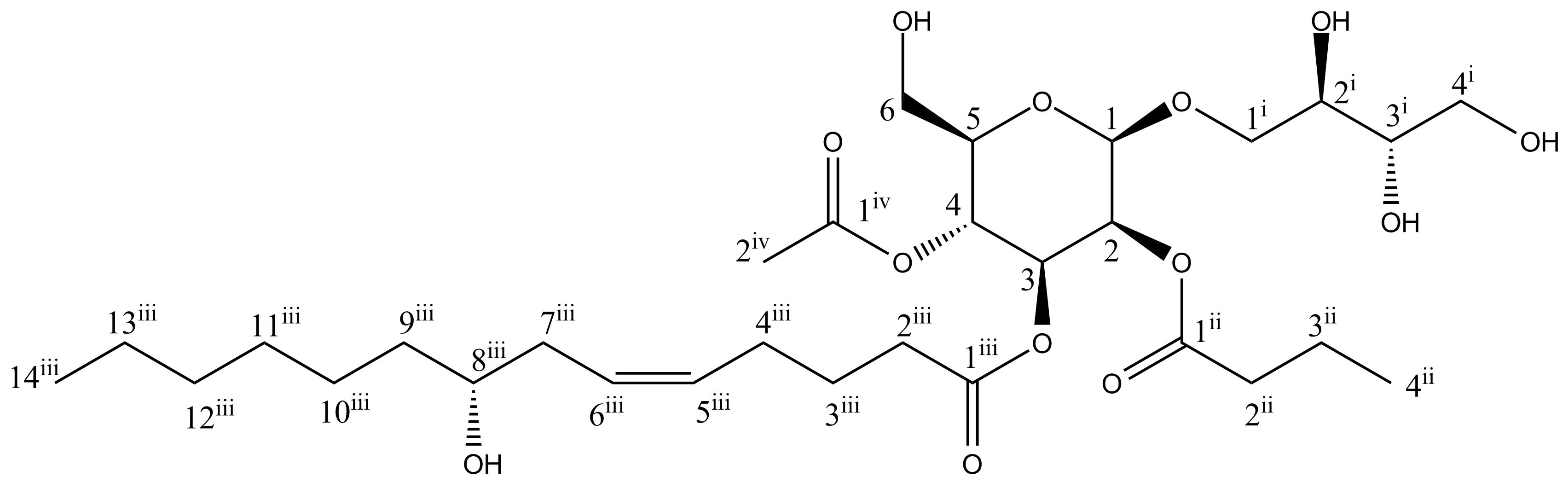 | | | | |
| Position | δ_C_ [ppm] | *multiplicity* | δ_H_ [ppm] | *multiplicity* | INT | J [Hz] |
| 1 | 98.7 | *d* | 4.91 | *br s* |  |  |
| 2 | 69.9 | *d* | 5.52 | *m** |  |  |
| 3 | 71.3 | *d* | 5.18 | *m** |  |  |
| 4 | 66.8 | *d* | 5.23 | *m* |  |  |
| 5 | 74.7 | *d* | 3.63 | *m* |  |  |
| 6 | 61.0 | *t* | 3.60  3.72 | *m**  *m** |  |  |
| 1^i^ | 71.9 | *t* | 3.68  4.10 | *m*  *m* |  |  |
| 2^i^ | 71.4 | *d* | 3.64 | *m* |  |  |
| 3^i^ | 72.7 | *d* | 3.61 | *m* |  |  |
| 4^i^ | 63.0 | *t* | 3.59  3.70 | *m**  *m** |  |  |
| 1^ii^ | 173.5 | *s* | - |  |  |  |
| 2^ii^ | 35.8 | *t* | 2.43 | *t* | 2H | 7.2 |
| 3^ii^ | 18.8 | *t* | 1.72 | *m* | 2H |  |
| 4^ii^ | 12.8 | *q* | 1.04 | *t* | 3H | 7.5 |
| 1^iii^ | 173.0 | *s* | - |  |  |  |
| 2^iii^ | 32.9 | *t* | 2.36 | *m* | 2H |  |
| 3^iii^ | 24.4 | *t* | 1.66 | *m* | 2H |  |
| 4^iii^ | 26.3 | *t* | 2.10 | *m* | 2H |  |
| 5^iii^ | 129.9 | *d* | 5.47 | *m** |  |  |
| 6^iii^ | 126.6 | *d* | 5.49 | *m** |  |  |
| 7^iii^ | 34.9 | *t* | 2.20 | *m* | 2H |  |
| **8^iii^** | **71.9** | ***d*** | **3.60** | ***m**** |  |  |
| 9^iii^ | 36.4 | *t* | 1.40  1.48 | *m**  *m** |  |  |
| 10^iii^ | 25.4 | *t* | 1.33  1.48 | *m**  *m** |  |  |
| 11^iii^ | 29.1 | *t* | 1.33 | *m* | 2H |  |
| 12^iii^ | 31.6 | *t* | 1.32 | *m* | 2H |  |
| 13^iii^ | 22.3 | *t* | 1.33 | *m* | 2H |  |
| 14^iii^ | 13.1 | *q* | 0.92 | *t* | 3H | 7.0 |
| 1^iv^ | 170.9 | *s* | - |  |  |  |
| 2^iv^ | 19.4 | *q* | 2.05 | *s* | 3H |  |

- * Overlapping signal
- Bruker TOPSPIN 4.0.9; 400 MHz (Solvent: CD_3_OD; δ_C_ = 48.0 ppm; δ_H_ = 3.33 ppm)

**Table S13** NMR analysis of *U. siamensis* MEL-B-C_4_-C_14:1-OH_-C_18:1_ (hydroxy-tri-MEL) produced from castor oil.

|  | | *U. siamensis* + castor oil | | | | |
| --- | --- | --- | --- | --- | --- | --- |
| **MEL type** | | MEL-B-C_4_-C_14:1-OH_-C_18:1_ | | | | |
| **Structure** | | 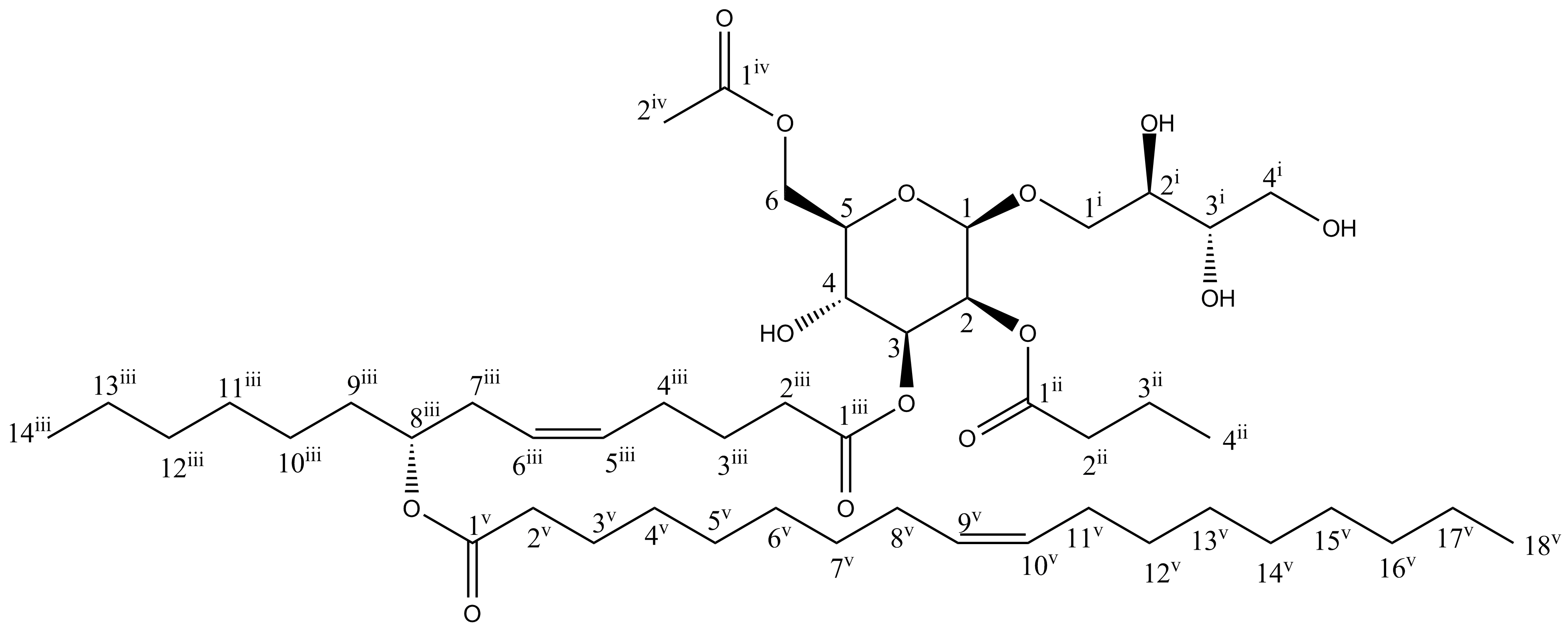 | | | | |
| Position | δ_C_ [ppm] | *multiplicity* | δ_H_ [ppm] | *multiplicity* | INT | J [Hz] |
| 1 | 99.1 | *d* | 4.86 | *br s* |  |  |
| 2 | 69.2 | *d* | 5.49 | *m** |  |  |
| 3 | 73.5 | *d* | 4.95 | *m** |  |  |
| 4 | 64.7 | *d* | 3.78 | *m** |  |  |
| 5 | 74.3 | *d* | 3.64 | *m** |  |  |
| 6 | 63.0 | *t* | 4.29  4.48 | *m**  *m** |  | 5.9, 12.0  1.8, 12.0 |
| 1^i^ | 71.5 | *t* | 3.70  4.05 | *m*  *m* |  |  |
| 2^i^ | 72.1 | *d* | 3.57 | *m* |  |  |
| 3^i^ | 70.8 | *d* | 3.68 | *m* |  |  |
| 4^i^ | 63.1 | *t* | 3.62  3.74 | *m**  *m** |  |  |
| 1^ii^ | 173.3 | *s* | - |  |  |  |
| 2^ii^ | 35.5 | *t* | 2.41 | *t* | 2H | 7.2 |
| 3^ii^ | 18.2 | *t* | 1.70 | *m* | 2H |  |
| 4^ii^ | 12.7 | *q* | 1.02 | *t* | 3H | 7.5 |
| 1^iii^ | 173.6 | *s* | - |  |  |  |
| 2^iii^ | 33.4 | *t* | 2.33 | *m* | 2H |  |
| 3^iii^ | 24.4 | *t* | 1.67 | *m* | 2H |  |
| 4^iii^ | 26.7 | *t* | 2.08 | *m* | 2H |  |
| 5^iii^ | 129.4 | *d* | 5.36 | *m** |  |  |
| 6^iii^ | 124.3 | *d* | 5.37 | *m** |  |  |
| 7^iii^ | 31.5 | *t* | 2.34 | *m* | 2H |  |
| **8^iii^** | **73.7** | ***d*** | **4.90** | ***m**** |  |  |
| 9^iii^ | 33.4 | *t* | 1.59 | *m** | 2H |  |
| 10^iii^ | 28.8 | *t* | 1.38  1.63 | *m**  *m** |  |  |
| 11^iii^ | 29.1 | *t* | 1.36 | *m* | 2H |  |
| 12^iii^ | 31.7 | *t* | 1.34 | *m* | 2H |  |
| 13^iii^ | 22.2 | *t* | 1.35 | *m* | 2H |  |
| 14^iii^ | 13.0 | *q* | 0.93 | *t* | 3H | 7.0 |
| 1^iv^ | 171.1 | *s* | - |  |  |  |
| 2^iv^ | 19.3 | *q* | 2.11 | *s* | 3H |  |
| 1^v^ | 173.9 | *s* | - |  |  |  |
| 2^v^ | 34.0 | *t* | 2.32 | *m* | 2H |  |
| 3^v^ | 24.8 | *t* | 1.63 | *m* | 2H |  |
| 4^v^ – 8^v^ | 29.2 | *t* | 1.34 | *m* | 10H |  |
| 9^v^ | 24.9 | *t* | 1.35 | *m* | 2H |  |
| 10^v^ | 26.8 | *t* | 2.10 | *m* | 2H |  |
| 11^v^ | 131.1 | *d* | 5.48 | *m* | 2H |  |
| 12^v^ | 132.1 | *d* | 5.50 | *m* | 2H |  |
| 13^v^ | 26.2 | *t* | 2.12 | *m* | 2H |  |
| 14^v^ – 15^v^ | 29.0 | *t* | 1.42 | *m* | 4H |  |
| 16^v^ | 31.7 | *t* | 1.38 | *m* | 2H |  |
| 17^v^ | 22.4 | *t* | 1.38 | *m* | 2H |  |
| 18^v^ | 12.9 | *q* | 0.93 | *t* | 3H | 7.0 |

- * Overlapping signal
- Bruker TOPSPIN 4.0.9; 400 MHz (Solvent: CD_3_OD; δ_C_ = 48.0 ppm; δ_H_ = 3.33 ppm)
